# Intensification of dairy production can increase the GHG mitigation potential of the land use sector in East Africa

**DOI:** 10.1111/gcb.14870

**Published:** 2019-11-19

**Authors:** Patric Brandt, Gabriel Yesuf, Martin Herold, Mariana C. Rufino

**Affiliations:** ^1^ Center for International Forestry Research (CIFOR) Nairobi Kenya; ^2^ Laboratory of Geo‐Information Science and Remote Sensing Wageningen University & Research Wageningen The Netherlands; ^3^ Lancaster Environment Centre Lancaster University Lancaster UK

**Keywords:** forest disturbance, greenhouse gas emissions, livestock grazing, LivSim, smallholder farming, sustainable intensification

## Abstract

Sub‐Saharan Africa (SSA) could face food shortages in the future because of its growing population. Agricultural expansion causes forest degradation in SSA through livestock grazing, reducing forest carbon (C) sinks and increasing greenhouse gas (GHG) emissions. Therefore, intensification should produce more food while reducing pressure on forests. This study assessed the potential for the dairy sector in Kenya to contribute to low‐emissions development by exploring three feeding scenarios. The analyses used empirical spatially explicit data, and a simulation model to quantify milk production, agricultural emissions and forest C loss due to grazing. The scenarios explored improvements in forage quality (Fo), feed conservation (Fe) and concentrate supplementation (Co): FoCo fed high‐quality Napier grass (*Pennisetum purpureum*), FeCo supplemented maize silage and FoFeCo a combination of Napier, silage and concentrates. Land shortages and forest C loss due to grazing were quantified with land requirements and feed availability around forests. All scenarios increased milk yields by 44%–51%, FoCo reduced GHG emission intensity from 2.4 ± 0.1 to 1.6 ± 0.1 kg CO_2_eq per kg milk, FeCo reduced it to 2.2 ± 0.1, whereas FoFeCo increased it to 2.7 ± 0.2 kg CO_2_eq per kg milk because of land use change emissions. Closing the yield gap of maize by increasing N fertilizer use reduced emission intensities by 17% due to reduced emissions from conversion of grazing land. FoCo was the only scenario that mitigated agricultural and forest emissions by reducing emission intensity by 33% and overall emissions by 2.5% showing that intensification of dairy in a low‐income country can increase milk yields without increasing emissions. There are, however, risks of C leakage if agricultural and forest policies are not aligned leading to loss of forest to produce concentrates. This approach will aid the assessment of the climate‐smartness of livestock production practices at the national level in East Africa.

## Graphical abstract

Richer cattle diets including cultivated grass and concentrates reduce GHG emission intensity by 33% and absolute emissions by 2.5%. Feeding silage and concentrates reduces intensity by 8%, whereas combining cultivated grass, silage and concentrates increases intensity by 12.5% due to land use change emissions. Closing the yield gap of maize by increasing N fertilizer use reduce intensities by 17% due to reduced emissions from conversion of grazing land.

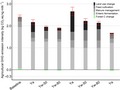

## 1 INTRODUCTION

Low agricultural productivity and population growth in Sub‐Saharan Africa (SSA) threaten current and future food security and increase the risk of degrading natural ecosystems (Grassi et al., 2017; Herrero et al., 2016). The majority of food in SSA is produced on farms with low productivity, stagnating crop yields due to nutrient‐depleted soils, and small farm sizes (Samberg, Gerber, Ramankutty, Herrero, & West, 2016; Sanchez, 2015). Food production on smallholder farms has to be intensified sustainably to reduce malnutrition and to adapt to erratic weather patterns and prolonged drought (Challinor, Koehler, Ramirez‐Villegas, Whitfield, & Das, 2016; van Ittersum et al., 2013). Climate‐smart agriculture (CSA) was conceived as a concept for agricultural systems to adapt to climate change, thereby mitigating anthropogenic impacts on the climate while safeguarding food security (FAO, 2013). Agriculture is the main cause of forest loss in SSA, resulting in reduced forest carbon (C) sinks and increased greenhouse gas (GHG) emissions (Carter et al., 2015). In addition to the conversion of forests into farm land, timber logging and fuelwood extraction, livestock grazing contributes to reduced forest C sinks by preventing tree regrowth (Brandt, Hamunyela, et al., 2018; Hosonuma et al., 2012; Pearson, Brown, Murray, & Sidman, 2017). Unlike in most high‐income countries, in SSA forest grazing is a common practice among cattle farmers and serves as an alternative source of feed when feed stocks on agricultural land are depleted (Sankhayan & Hofstad, 2001; Schiere, Ibrahim, & Keulen, 2002). Together, these anthropogenic activities modify forest structure, reduce C sequestration, affect water and nutrient cycling (Lawrence & Vandecar, 2015), and biodiversity (Barlow et al., 2016), which ultimately have a negative feedback on agricultural production.

To address these increasing pressures, several SSA countries are developing policies and instruments that combine elements of CSA and development targets for the agricultural sector. Kenya, as a good example of such countries, has recently developed a national CSA strategy, which aims to transform the country's agricultural sector towards a climate‐smart food production system. Agriculture is not only Kenya's economic backbone, but it also contributes approximately 40% of the country's GHG emissions budget. About 90% of the agricultural emissions are associated with livestock production (Government of Kenya, 2015a). As part of its ambitious economic development plan, Kenya seeks to develop its dairy sector to be able to meet the increasing demand for milk driven by a booming urban population (Government of Kenya, 2010). Dairy production engages approximately two million smallholder farmers, who contribute about 80% of the total milk production in Kenya (Udo, Weiler, Modupeore, Viets, & Oosting, 2016). Increasing the production by larger herds would raise the demand for feeds, increase GHG emissions from enteric fermentation, animal manure, and augment soil emissions from feed production and the more intensive utilization of pastures. A plan to increase the country's milk production is constrained by the current low yields of feed crops, small farm sizes, and insufficient arable land, which results in increased pressure on natural forests and the risk of forest loss (Bosire et al., 2016; Brandt, Hamunyela, et al., 2018). To develop dairy production in accordance with CSA objectives, the dairy sector will have to intensify the milk production sustainably so that the increase in production does not lead to higher demands for agricultural land and to the resulting expansion into natural ecosystems.

The Nationally Determined Contribution (NDC) and the dairy master plan of Kenya define specific targets for climate change mitigation and for the development of the livestock sector. According to these national policies, the increase in total GHG emissions in Kenya has to be lowered by 30% relative to projected business‐as‐usual emissions between 2010 and 2030 (Government of Kenya, 2015b). Within the same period, milk yields per cow should increase by 150% to ensure that local dairy production meets the increased requirements for food and nutrition due to population growth (Government of Kenya, 2010). Furthermore, a newly developed Nationally Appropriate Mitigation Action (NAMA) for the dairy sector in Kenya defines a low‐emission development pathway, which aims to increase on‐farm productivity by promoting the adoption of high‐quality feeds (Government of Kenya, 2017).

Forest degradation is the largest component (75%) of the forest emissions in Kenya, where deforestation rates have been around 35,000 ha/year in the last three decades (Carter, Herold, et al., 2018; Pearson et al., 2017). In a recent analysis, Brandt, Herold, and Rufino (2018) reported potential synergies between milk yield increases and GHG mitigation benefits on agricultural land to be realized by improving the quality of dairy feeds. In addition, intensified smallholder dairy farms located close to forests were associated with a reduced risk of local forest degradation (Brandt, Hamunyela, et al., 2018). The analyses showed that changes in livestock diets may require the conversion of grazing land to cultivate more nutritious feeds, which would cause GHG emissions from land use change (LUC) and reduce the effectiveness of the feed improvements for climate change mitigation (Brandt, Herold, et al., 2018). Promoting dairy production in regions without agricultural land available for feed cultivation could, therefore, increase the risk of C leakage as farmers may use adjacent forests for grazing. In these cases, closing the yield gap of feed crops may reduce the demand for additional land and, thus, alleviate the pressure on forests. To date, there are no assessments that integrate the effects of dairy intensification and GHG mitigation on forests in SSA. However, the integration of these land use sectors is crucial for effective CSA targeting and planning with the added value of preventing C leakage. This study was designed to answer two main questions: (a) Can feed improvement strategies reduce total GHG emissions from agricultural production? and (b) Can emission intensities from dairy production be reduced? We addressed these questions by exploring the potentials of improved dairy feeds, including closing the yield gap of fodder maize and decreasing forest C loss due to livestock grazing in the dairy production region of Kenya. We used empirical data from Kenya's dairy production region, and the livestock production model LivSim (Rufino et al., 2009) to calculate milk yields and GHG emissions for three different intensification scenarios. Remote‐sensing data were used to quantify forest C change and to approximate, for the first time, an estimation of forest C loss related to livestock grazing. The scenarios considered in this study are aligned with current policy objectives of dairy intensification and mitigation of GHG emissions in the agricultural and forestry sectors.

## 2 MATERIAL AND METHODS

### 2.1 Study area

The intensive dairy sector is located in the Central and Western highlands of Kenya covering about 65,000 km^2^ and characterized by smallholder crop–livestock production (Herrero et al., 2014). The area shows the highest densities of human and livestock populations throughout Kenya (Imo, 2012), and produces most of the milk that is marketed in the country. This region is also home to the last Afromontane forests, often called ‘the water towers’ because of their important role supplying water to urban centres (Jacobs, Breuer, Butterbach‐Bahl, Pelster, & Rufino, 2017). These forests include the Aberdare range, the Cherangani Hills, the Mau Forest, Mount Elgon and the Mount Kenya Forest. All these forests are under enormous pressure from population growth and forest degradation due to the unsustainable use of forest resources (Drigo, Bailis, Ghilardi, & Masera, 2015; Imo, 2012).

### 2.2 Analytical framework

**Figure 1:**
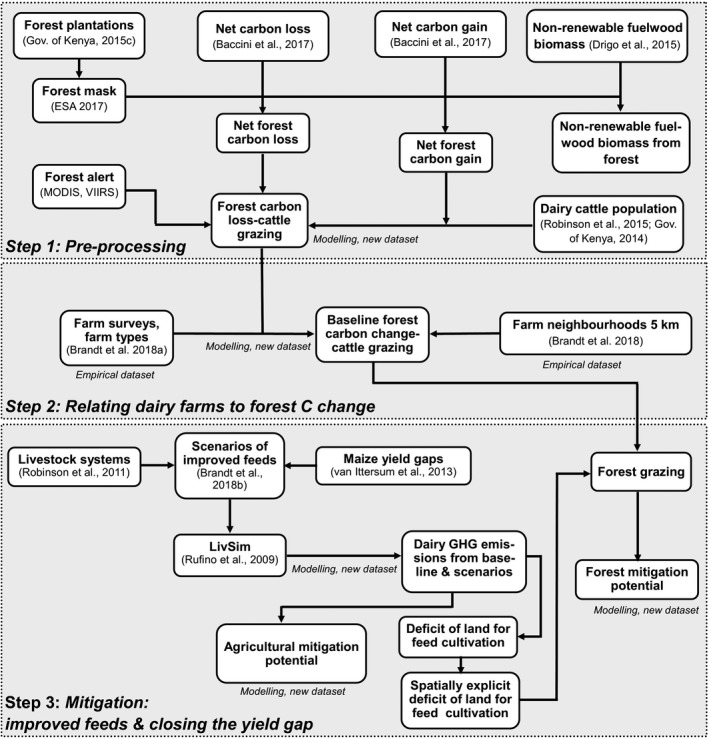
Calculation of mitigation potential on agricultural land and forests for feed improvement scenarios, which include closing the yield gap of maize. Carbon (C) loss‐grazing = C loss due to dairy cattle grazing in forests, Carbon change‐grazing = Forest C change, which includes net forest C gain minus the C loss fraction due to dairy cattle grazing in forests, LivSim = livestock simulation model. Publically available data sets and models include references, empirical and new data sets are indicated using italics

Three steps were followed in the analyses (Figure 1): First, spatially explicit data on net forest C loss and gain were preprocessed. To derive forest C loss due to dairy cattle grazing, forest C loss from forest fires was excluded and fuelwood extraction subtracted from net forest C loss. Second, to quantify the relationship between smallholder farming practices and forest C change, farm indicators and farm types derived from a farm survey conducted by Brandt, Herold, et al. (2018) were related to net forest C loss, gain and change. Third, the livestock simulation model LivSim was run to compute spatially explicit data on milk production, agricultural GHG emissions from dairy production and the requirement of land to produce feeds. The composition of feeds in the baseline and three feed scenarios reflect typical diets for dairy cows in Kenya. The scenarios include (a) improving forage quality (FoCo) by supplementing larger quantities of Napier grass (*Pennisetum purpureum* Schumach.) with concentrates; (b) using feed conservation (FeCo) by producing maize silage and feeding concentrates, closing the yield gap of fodder maize; and (c) a combination of Napier grass, maize silage and concentrates (FoFeCo). For these scenarios, the amount of additional land required to cultivate these high‐quality feeds was computed to estimate deficits of agricultural land for each pixel. Data sets of different resolution were resampled to a pixel resolution of 1 km^2^. Subsequently, the deficit of agricultural land to produce feeds for dairy cattle around forests was linked to forest grazing and C loss for each scenario to be able to quantify mitigation potential.

### 2.3 Preprocessing (Step 1)

This step produced a new data set that quantified the forest C losses due to grazing cattle. Spatially explicit data sets of the changes in above‐ground biomass between 2003 and 2014 were used to quantify annual forest C changes (Baccini et al., 2017). These data sets include net gains (C gain) and losses of C (C loss) at a pixel resolution of 463 × 463 m and resampled to 1 km^2^ (Figure 1, step 1). A forest mask was applied to restrict the C change data to forests in 2016 to derive net forest C loss and net forest C gain. The forest mask was based on the land cover data set of Africa with a pixel resolution of 20 × 20 m (ESA, 2017). In addition, a data set of tree plantations from the Government of Kenya (2015c) was used to limit the forest mask to natural forests.

Forest wildfires emit substantial amounts of C (Hurteau, Koch, & Hungate, 2008), and leave open forests accessible for opportunistic livestock grazing. However, the C loss due to forest fires can neither be attributed to the presence of cattle in forests nor can this C loss be mitigated through improvements of cattle feeding. Therefore, pixels that indicate burnt forest between 2003 and 2014 were excluded from forest C loss (Figure 1, step 1) using daily fire alert data from the ‘Moderate Resolution Imaging Spectroradiometer’ (MCD14ML; Giglio, 2015) and the ‘Visible Infrared Imaging Radiometer Suite’ (Schroeder, Oliva, Giglio, & Csiszar, 2014).

Fuelwood consumption in Kenya exceeds the capacity of forests to regrow and is responsible for about one‐third of the total forest emissions (Bailis, Drigo, Ghilardi, & Masera, 2015; Pearson et al., 2017). To account for this source of forest C loss, a spatially explicit data set of nonrenewable biomass (NRB) harvested annually as fuelwood was used at a pixel resolution of 100 × 100 m (Drigo et al., 2015). The NRB data set was subtracted from the net forest C loss data after restricting it to forests using the same forest mask applied previously to derive forest C loss‐cattle grazing (Figure 1, step 1). The proportion of dairy cattle was calculated by excluding beef cattle using county‐level data on cattle types (Government of Kenya, 2014). The forest ‘C loss‐cattle’ data was multiplied by the proportion of dairy cattle to calculate forest C loss that could be attributed to dairy cattle. The estimate of forest C loss due to grazing cattle is the first approximation of this C loss component and is based on our previous empirical work with observations of cattle grazing in the forest (Brandt, Hamunyela, et al., 2018). To our knowledge there are no other spatially explicit data available that quantify the effects of livestock on carbon loss in East African montane forests.

To account for the propagation of uncertainties from input data sets such as net forest C loss and forest NRB which were used to quantify forest C loss from cattle grazing, we used the method by Lee and Forthofer (2006) and is expressed in Equation (1).

(1) $$\begin{array}{cc} \text{var}(\text{forest}\;C\;\text{loss}\;-\;\text{cattle}\;\text{grazing}) & \;=\;\text{var}(\text{net}\;\text{forest}\;C\;\text{loss})+\text{var}(\text{forest}\;\text{NRB})\\ & \;-2\;\times \;\text{cov}(\text{net}\;\text{forest}\;C\;\text{loss,}\;\text{forest}\;\text{NRB}), \end{array}$$

where var(forest C loss‐cattle grazing) is the variance associated with C loss from forest grazing, var(net forest C loss) is the variance of net forest C losses, var(forest NRB) is the variance of fuelwood extraction and cov(net forest C loss, forest NRB) is the covariance of net forest C loss and loss from fuelwood extraction.

Relative standard deviations (*SD*) reported in Baccini et al. (2017) and Bailis et al. (2015) were used to quantify input variance and to estimate propagated uncertainties.

### 2.4 Spatial relationship between dairy farms and forest C change (Step 2)

The effects of smallholder dairy farms on forest C change were based on empirical relationships established for the study area by Brandt, Hamunyela, et al. (2018). These analyses linked farming practices and farm characteristics to forest disturbance calculated from a remote‐sensing‐based time‐series analysis, and validated in the field (Brandt, Hamunyela, et al., 2018). The empirical data were obtained through a farm survey conducted in 2016 sampling 216 smallholder farms, located in close vicinity to forests. Farm indicators included fuelwood extraction, milk yields, feed types in the cattle diet (such as grass from on‐farm pastures, fodder crops and dairy concentrate), farm area allocated to fodder crops and pastures, total farm size, total numbers of cattle and number of improved dairy cattle. Improved dairy cattle are cross breeds between *Bos taurus* and *Bos indicus*, most commonly using artificial insemination with semen from Friesian bulls. Using these data, farms were clustered into three farm types: ‘small and resource‐poor farms’, ‘large and inefficient farms’ and ‘intensified farms’. The results indicated that farms with more cattle and lower milk yields were associated with stronger forest disturbance effects and that farms, which used improved diets and attained higher milk yields caused less forest disturbance.

Farm indicators and farm types were linked to the forest C change data from remote sensing using circular buffers around farm centroids (Figure 1, step 2) to test whether similar forest disturbance and farm characteristics were detectable. A radius (*r*) of 5 km was selected for these buffers or ‘farm neighbourhoods’, because this radius determined the maximum neighbourhood size for which farm indicators and farm types were significantly correlated with forest disturbance (Brandt, Hamunyela, et al., 2018). Forest C change included only the C loss correlated with dairy cattle (i.e. forest C change‐cattle grazing). Farm indicators were correlated with net forest C loss, net forest C gain, forest NRB, forest C loss‐cattle grazing and forest C change‐cattle grazing. Collinearity between the selected farm indicators was previously checked by Brandt, Hamunyela, et al. (2018) and those that were highly correlated (Spearman's *ρ* ≥ 0.7) were excluded. Differences between farm types were tested using the nonparametric pairwise Wilcoxon rank sum.

### 2.5 Quantifying the mitigation potential of dairy feed improvements (Step 3)

#### 2.5.1 Livestock modelling and feed scenarios

The livestock production model LivSim (Rufino et al., 2009) was used to quantify milk production and agricultural GHG emissions from smallholder dairy production. GHG emissions were quantified by following IPCC tier 2 methodology (IPCC, 2006) and included methane (CH_4_) emissions from enteric fermentation, CH_4_ emissions from manure management, direct and indirect nitrous oxide (N_2_O) emissions from manure management, direct and indirect N_2_O emissions from managed soils, including fertilizer application and N_2_O and carbon dioxide (CO_2_) emissions from LUC. Under each scenario, manure management varied depending on the amount of cropland required to cultivate Napier grass and fodder maize. For instance, in scenarios with reduced grazing, (FoCo and FoFeCo) the amount of manure dropped onto pastures was reduced and more manure was stored on heaps for further use as organic fertilizer. The approach accounted explicitly for emissions from synthetic fertilizer application, and did not include emissions from the production and transport of fertilizers due to lack of data.

Improving diets often requires the cultivation of energy and protein‐dense feeds with high digestibility that can increase milk yields (Hristov et al., 2013). Producing these feeds may require additional cropland and may cause the conversion of grazing or forest land. This analysis accounted for conversion of grazing land to croplands, and to estimate the impacts of concentrate production, we calculated emissions from their production as the land footprint for each scenario. To calculate the emissions from concentrate production we used average composition and the emission factor reported by Weiler, Udo, Viets, Crane, and De (2014). For the land footprint we used yields for the different ingredients from FAOStat (2019). Milk yield and GHG emissions were computed by simulating dairy cows over a lifetime of 13 years (Rufino et al., 2009; Tables S1 and S2). Model outputs were upscaled following the method of Brandt, Herold, et al. (2018) and represented in Figure S1. Subsequently, model outputs were mapped using spatially explicit data on livestock production systems (LPS), cattle density (Robinson et al., 2011, 2014) and dairy herd composition (Bebe, Udo, & Thorpe, 2002; Government of Kenya, 2014).

Milk yields and GHG emissions were calculated for the baseline and scenarios (Figure 1, step 3). The baseline represented a typical diet for smallholder dairy cattle in Kenya with a large proportion of low‐quality grass and crop residues (Table S3). The analyses focused on scenarios reported in Brandt, Herold, et al. (2018) as follows: (a) improving forage quality by adding more Napier grass; (b) conserving feed as maize silage; and (c) increasing the supplementation of dairy concentrates. These strategies were combined into the three scenarios: ‘forage quality and concentrate supplementation’ (FoCo), ‘feed conservation and concentrate supplementation’ (FeCo) and ‘forage quality, feed conservation and concentrate supplementation’ (FoFeCo). The baseline feeds for each LPS were replaced by 25% and 50% higher‐quality feeds (on a dry matter basis) and rations of concentrate were increased to 3 and 6 kg/day during the 150 day of early lactation representing medium intensification and high intensification levels respectively (Table S3). Changes in feeds relative to the baseline for each LPS are shown in Figure S2. The choice of scenarios was based on their mitigation potentials: (a) FoFeCo with low potential at high intensification; (b) FeCo with medium potential at medium intensification; and (c) FoCo with high potential at medium intensification level. The mitigation potential was assessed through milk production and GHG emission intensity, including LUC but avoiding deforestation. The ranges of GHG emission parameters were sampled using Latin hypercube sampling (LHS; Xu et al., 2005) to estimate overall emission uncertainties of the baseline. Each parameter was sampled separately through LHS while all others were kept at their mean values. Emission uncertainties of the scenarios were estimated one parameter at a time, sampling at the minimum and the maximum of the parameter ranges.

#### 2.5.2 Closing yield gaps of maize

For each scenario, the cultivation of Napier grass and maize requires a certain amount of land. Brandt, Herold, et al. (2018) reported that scenarios which include maize silage require additional cropland to grow maize to prevent detrimental effects on food security since maize is mainly used for human consumption. In the highlands of Kenya, the yield gap of maize ranges between 30% and 82% suggesting a high potential to intensify maize production (van Ittersum et al., 2013). Closing the yield gap of maize using fertilizers would reduce the additional land demand calculated in the scenarios. Carbon emissions from LUC would be lowered at the expense of N_2_O emissions from soils due to increased rates of fertilizer application.

For this study, we selected the water‐limited yield potential (Yw) as the benchmark indicator for maize accounting for yield‐limiting factors such as water supply, soil properties (e.g. water holding capacity) and topography (e.g. runoff). The actual maize yields of the baseline were increased to reach 50% and 80%. Farm yields often reach a saddle point around 80% of Yw, where it is not feasible for farmers to increase any further (van Ittersum et al., 2013). Actual yields (Ya) of maize cultivated in the Kenyan highlands were obtained from Castellanos‐Navarrete, Tittonell, Rufino, and Giller (2015), Monfreda, Ramankutty, and Foley (2008), and Weiler et al. (2014). Data on Yw of maize and the nitrogen (N) input required to realize Yw at 50% and 80% in Kenya were obtained from the Global Yield Gap Atlas. These estimates are based on agroclimatic zones used to upscale location‐specific yield estimates from crop simulation modelling (van Wart, Kersebaum, Peng, Milner, & Cassman, 2013; van Wart, van Bussel, et al., 2013). Yw and N input were linked to the LPS classification used in this study to upscale milk yield and GHG emissions. N input is the nitrogen requirement to achieve a target maize yield of either 50% or 80% water‐limited yield potential. The value of N input was increased by a factor to account for nitrogen use efficiencies of 33% for fertilizer N and 20% for manure N and used as an approximation of the crop's nitrogen uptake in above‐ground biomass, which in Kenya can be between 69 and 185 kg N/ha (Table S4; ten Berge et al., 2019). FeCo and FoFeCo which included maize silage were calculated for Ya, Yw at 50% and 80% (Figure 1, step 3).

#### 2.5.3 Land requirements to feed dairy cattle

Land requirements were calculated for each scenario by comparing the extent of grazing land and land demand to cultivate feeds per pixel using the R library raster (v. 2.5; Hijmans, 2016; R Core Team, 2016). Only existing grazing lands were assumed to be available to cultivate additional Napier grass and maize, and we consequently calculated emissions from LUC using emission factors from Don, Schumacher, and Freibauer (2011). Demands for additional cropland were quantified based on the actual yields per feed type, water‐limited yield potentials of maize, crop‐specific feed intake per dairy cow (see Table S5) and the density of dairy cattle per 1 × 1 km pixel extracted from Robinson et al. (2014). Available land was quantified using a spatially explicit data set on grazing land (Velthuizen et al., 2007). Each pixel where the demand for cropland to produce feeds exceeded the extent of grazing land available was labelled as a pixel with land deficit. Spatially explicit polygons of land deficit were created for each scenario (Figure 1, step 3; Supporting Information section 4). On‐farm land requirements to produce concentrate ingredients were not included because dairy farmers purchase concentrates from the market and some ingredients are imported from outside Kenya (Weiler et al., 2014). However, to get an indication of the potential impact of concentrate production on indirect LUC, the land footprint associated with the annual amount of concentrate required was calculated using crop yields from FAOStat (2019). Although deforestation in Kenya is limited to about 35,000 ha/year, we estimated the C emissions that would result from a worst‐case scenario of conversion of forests to cropland to produce the concentrate ingredients, using an emission factor of 112.7 ± 3.9 Mg CO_2_eq/ha from Carter, Herold, et al. (2018).

The data sets on forest C loss‐cattle grazing, forest C gain and the polygons of agricultural land deficit were used to link forest C change to the production of feed crops on agricultural land. All pixels with forest C loss‐cattle grazing, and forest C gain were assumed to be the baseline ‘forest C change’ to the grazing of dairy cattle. In the scenarios, it was assumed that the deficit of land to produce feed crops cause grazing in adjacent forests. Brandt, Hamunyela, et al. (2018) reported negative effects of livestock management on forest disturbance for farm neighbourhoods with a radius of 5 km. Therefore, polygons of land deficit were buffered using this distance. For each scenario, pixels of forest C loss‐cattle grazing that intersected with polygons of land deficit represented forest C losses due to grazing. Finally, the sum of ‘forest C change’ due to grazing was calculated for each scenario and compared to the baseline to quantify the mitigation potential in forests (Figure 1, step 3).

## 3 RESULTS

### 3.1 Smallholder farms and forest C change

**Figure 2:**
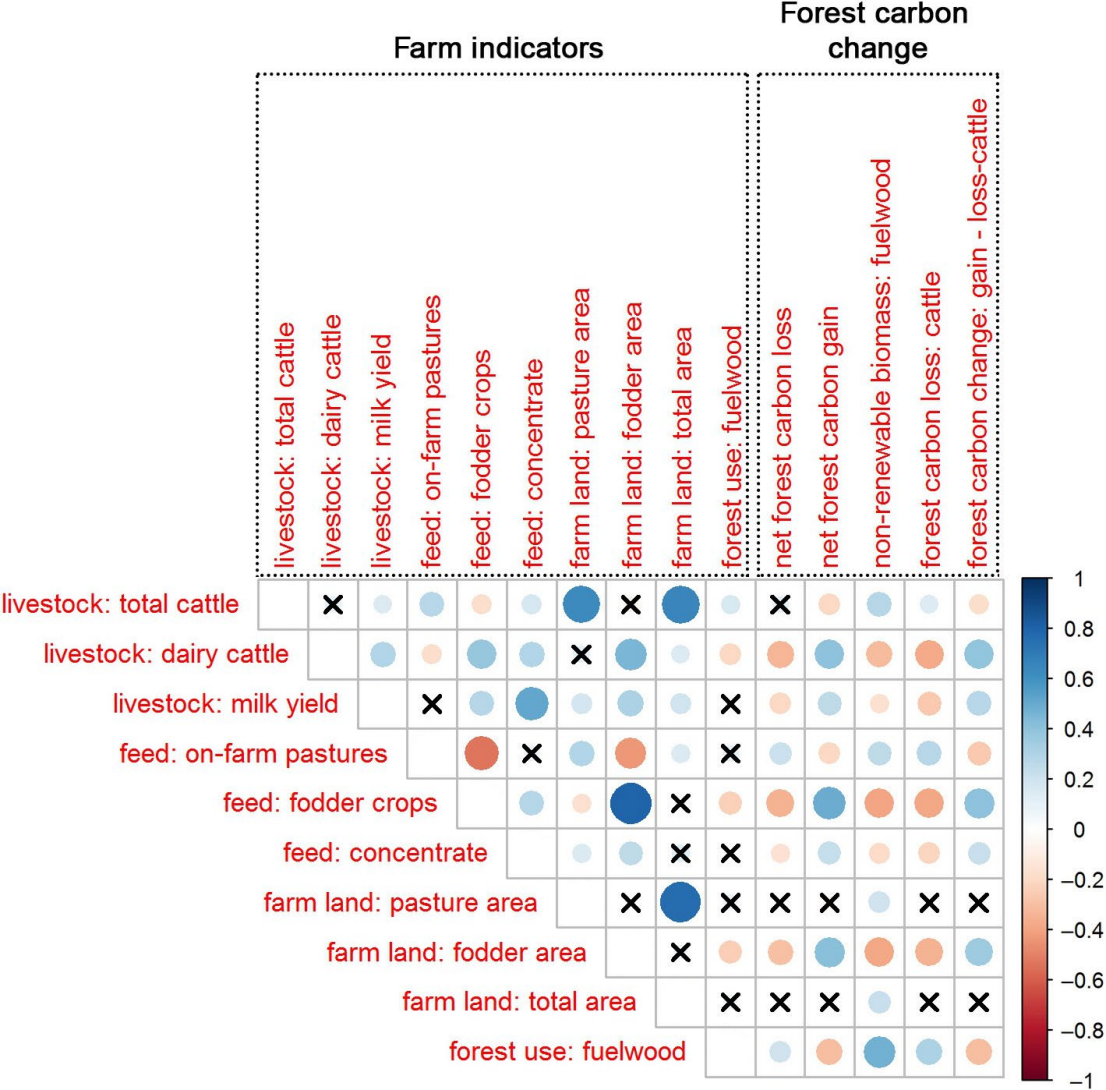
Correlation between farm indicators and forest carbon C change variables: net forest C loss, net forest C gain, nonrenewable biomass use related to fuelwood harvest in forests (forest NRB), forest C loss‐cattle grazing and forest C change‐cattle grazing, which includes forest C gain and the forest C loss‐cattle grazing. Circle size indicates the strength of significant correlations. Blue = positive correlation, red = negative correlation

The empirical analysis of the farm data collected in the study region showed that number of cattle on farm was positively correlated with forest C loss due to grazing (*ρ* = 0.15; *p* < .05) and negatively correlated with the forest C change‐cattle grazing (*ρ* = −0.17; *p* < .05; Figure 2). The number of improved dairy cattle per farm and milk yield were negatively correlated with forest C loss due to grazing (*ρ* = −0.37, −0.26; *p* < .001) and positively correlated with C change due to grazing (*ρ* = 0.39, 0.27; *p* < .001). The farm indicators of feed intensification (proportion of fodder crops in the diet, supplementation of concentrates and farm area allocated to fodder crops) were negatively correlated with forest C loss due to grazing (*ρ* = −0.39, −0.21, −0.34; *p* < .001) and positively correlated with forest C change (*ρ* = 0.41, 0.22, 0.36; *p* < .001). Fuelwood extraction was positively correlated with NRB harvest (forest NRB; *ρ* = 0.47; *p* < .001).

**Figure 3:**
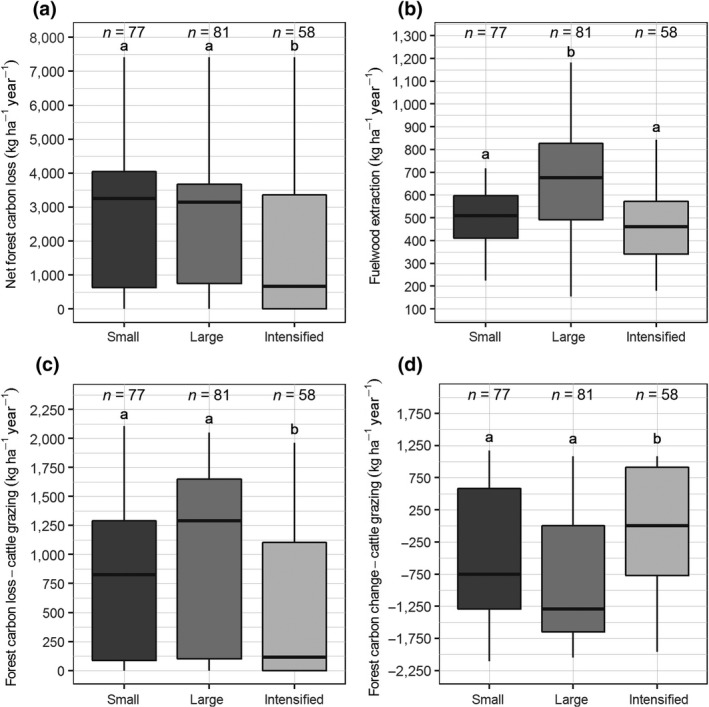
(a) Net forest C loss, (b) forest C loss from fuelwood extraction (forest nonrenewable biomass), (c) forest C loss from cattle grazing, and (d) forest C change due to cattle grazing, which is forest C gain minus the forest C loss‐cattle grazing for three farm types. Farm types are: small = small and resource‐poor farms, large = large and inefficient farms, intensified = intensified farms. Negative values in panel d indicate forest C loss. Different letters above whiskers indicate significant differences between farm types using pairwise Wilcoxon rank sum tests (*p*‐values were corrected for multiple testing)

Farm types had different effects on net forest C loss, forest NRB, forest C loss‐cattle grazing and forest C change‐cattle grazing (Figure 3). The results reported here refer to the area of influence of the dairy farms, empirically determined to be 5 km from the forest edge. Intensified farms were associated with significantly less forest C loss, less forest C loss‐cattle grazing and higher forest C change‐cattle grazing (means = 1,676.4, 512.0, and −54.6 kg C ha^−1^ year^−1^ respectively) than small and resource‐poor farms (means = 2,476.5, 855.5, and −565.8 kg C ha^−1^ year^−1^ respectively) and large and inefficient farms (means = 2,564.6, 980.6, and −842.0 kg C ha^−1^ year^−1^ respectively; *p* < .05; Figure 3a,c,d). Large and inefficient farms were associated with significantly higher forest NRB (mean = 656.2 kg C ha^−1^ year^−1^) than small and resource‐poor farms (mean = 501.0 kg C ha^−1^ year^−1^) and intensified farms (mean = 463.1 kg C ha^−1^ year^−1^; *p* < .05; Figure 3b).

### 3.2 Agricultural and forest mitigation potentials

#### 3.2.1 Agricultural GHG emissions

**Figure 4:**
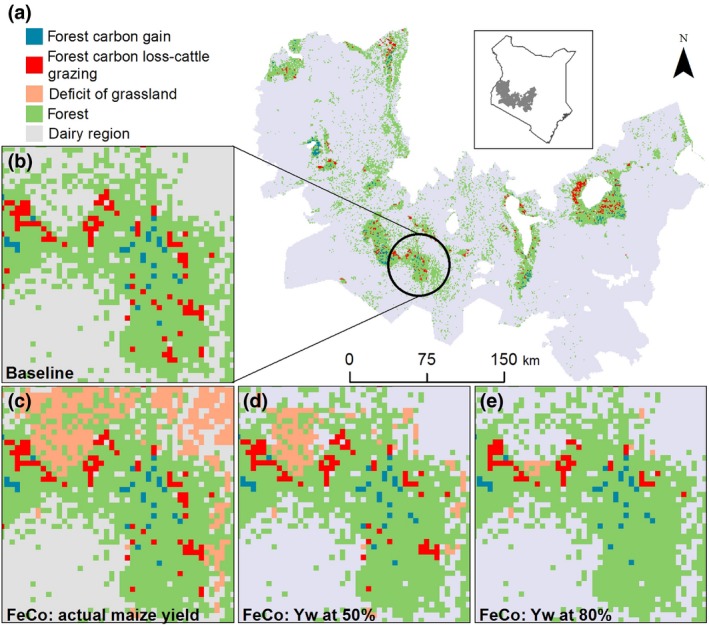
Forest C loss due to dairy cattle and forest C gain. (a) Baseline forest C loss‐cattle grazing and forest C gain for the dairy region of Kenya. (b) Baseline forest C loss‐cattle grazing and forest C gain for the Maasai Mau Forest. (c–e) Forest C loss‐cattle grazing due to the deficit of grassland for the scenario FeCo, which combines feed conservation based on maize silage and concentrate supplementation. (c) Deficit of arable land and the forest C loss‐cattle grazing with actual maize yields (Ya). Achieving water‐limited yield potential (Yw) of maize at 50% (d) and 80% (e) may reduce the deficit of arable land by 7,729 and 14,158 ha respectively

All forests located in the study area were affected by C losses and gains between 2003 and 2014, losing 781.6 Mg and on average 8.9 kg CO_2_eq ha^−1^ year^‐1^ due to the grazing dairy cattle (Figure 4a,b). Changes in feeding, and closing the yield gap of maize used for silage production could reduce the amount of forest C loss. This effect is shown for the scenario FeCo, which combined feed conservation based on maize silage and concentrate supplementation (Figure 4c). However, deficit of arable land in the vicinity of forests to cultivate maize would lead to forest C loss due to cattle grazing to cover the feed shortage. Closing the yield gap of maize may reduce the amount of land required to grow additional maize and, therefore, could alleviate the land deficit as shown for the Maasai Mau Forest region (Figure 4c–e).

**Figure 5:**
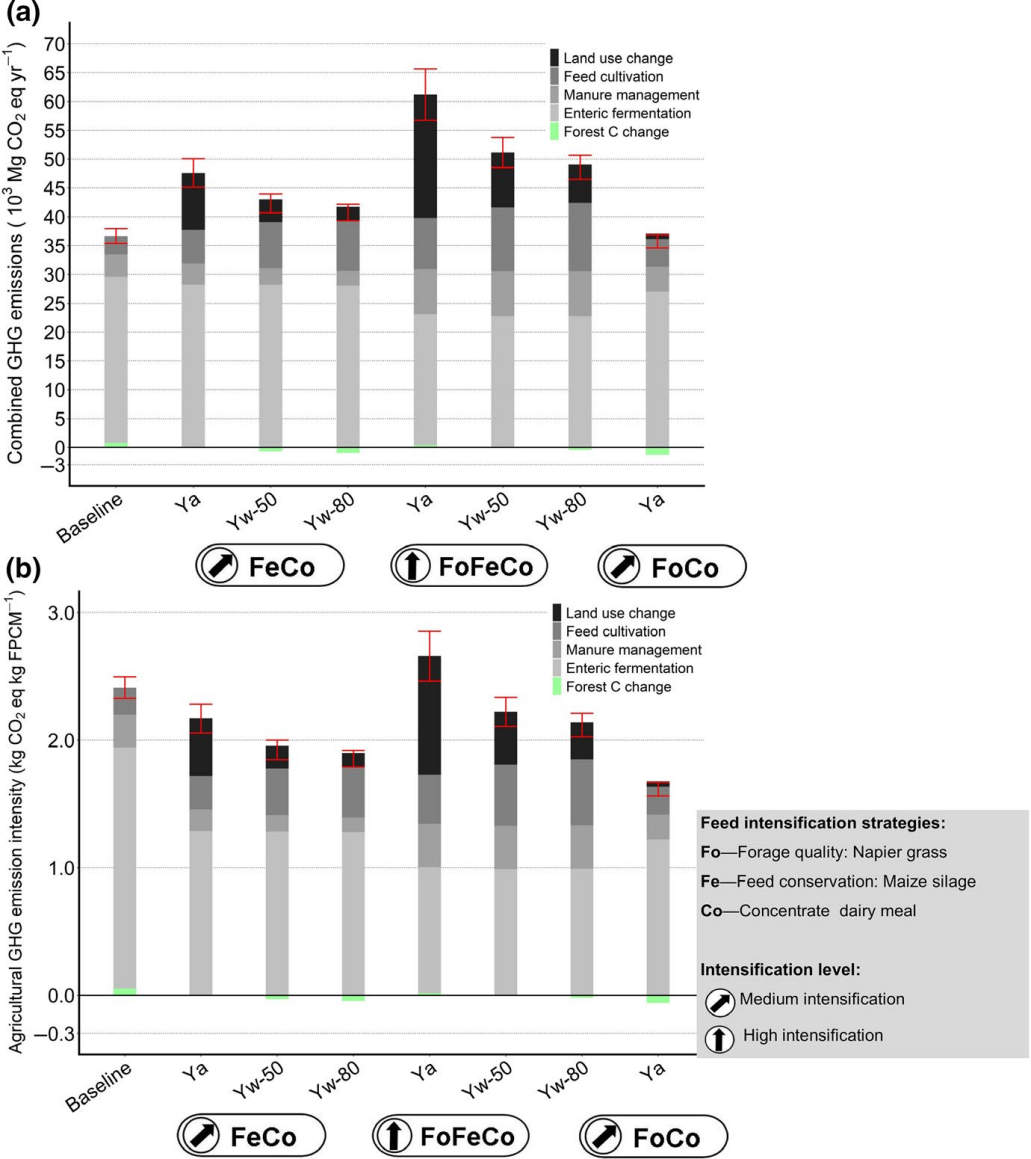
Agricultural greenhouse gas (GHG) emissions and forest carbon C change related to dairy production. (a) Combined GHG emissions including those from forest C change due to cattle grazing; (b) GHG emission intensity per kg fat and protein corrected milk (FPCM). Bars indicate baseline and three scenarios at medium (FoCo, FeCo) or high (FoFeCo) intensification levels. FoCo and FoFeCo included maize silage, where we show actual maize yields (Ya) and Yields at 50% (Yw‐50) and 80% (Yw‐80) of water‐limited yield potentials (Yw). Error bars indicate relative *SD*s

**Figure 6:**
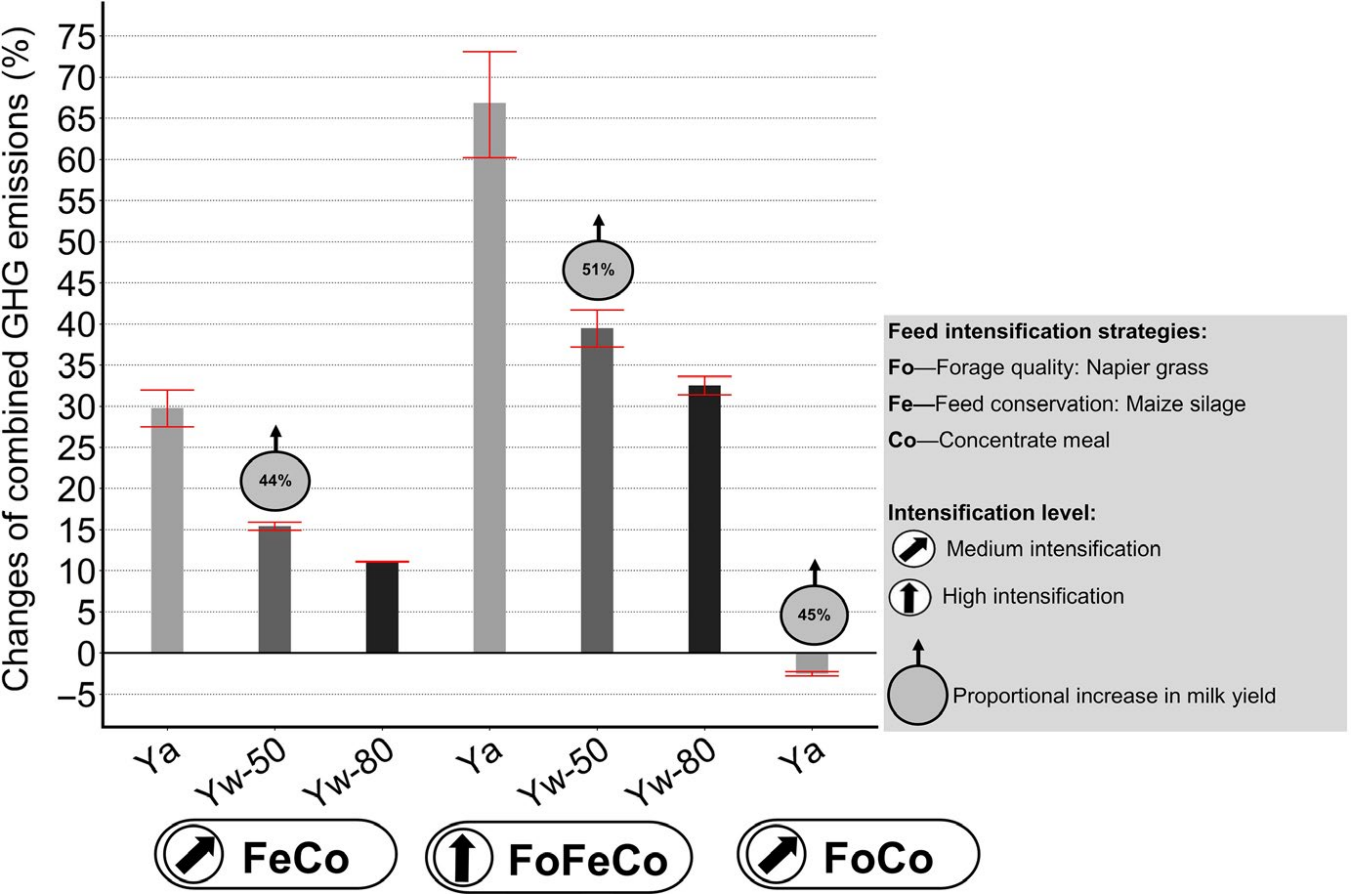
Changes in total agricultural greenhouse gas (GHG) emissions and forest carbon (C) change combined, which is forest C gain minus C loss due to cattle grazing. Bars show changes in GHG emissions for different scenarios and the baseline. Ya = actual baseline yields of maize, Yw‐50 = Yw realized at 50%, Yw‐80 = Yw realized at 80%. Bubbles indicate the percentage milk yield increase for each scenario

Across the study area, the scenarios increased total agricultural GHG emissions in relation to the baseline by 3.2%–69.4% (±2.8–6.5; Figure 5a). The lowest increase of GHG emissions was for the FoCo scenario. The highest increase in emissions was for the FoFeCo scenario and with actual maize yields (baseline Ya), although GHG emissions from enteric fermentation were reduced by 1.9%–21.1%. The FoCo and FoFeCo scenarios had the lowest and highest effect on reducing emissions from enteric fermentation. Emissions from manure management increased by up to 100% for the FoFeCo scenario. More N was excreted by cattle when the proportion of Napier grass in the cattle diet increased, which led to higher N_2_O emissions. GHG emissions from soils used to produce cattle feeds increased by 48.3%–266.5%. The FoFeCo scenario, which included maize to produce silage and a water‐limited yield potential of Yw‐80 led to highest increases in feed‐related emissions (Figures 5a and 6) due to high fertilizer N application rates of up to 108.2–167.9 kg N/ha required to achieve higher yields. The scenarios that included silage (FeCo and FoFeCo) also had higher emissions from LUC compared with the FoCo scenario. Maize production required more land than Napier grass increasing LUC emissions from the conversion of grazing lands into cropland (Figure 5a). However, by increasing maize yield (i.e. Ya to Yw‐80) LUC emissions from FeCo and FoFeCo scenarios were reduced by 69.0%–75.3%. The reduction of emissions from LUC was 2.6–4.9 times higher than the increase of emissions from additional fertilizer N. Despite the reduction of emissions from enteric fermentation and LUC by closing the yield gap of maize, none of the feed scenarios would achieve a net GHG reduction from agricultural land. However, reductions in GHG emission intensities from forest C change would be achieved under FoCo, and FeCo and FoFeCo when closing yield gaps (Tables S7 and S8).

#### 3.2.2 Land footprint

The land footprint analysis indicated that under current average yields and for the medium intensification scenarios (FoCo and FeCo) over 500,000 ha would be required for concentrate production, while under the high intensification scenario (FoFeCo) land requirement would be approximately 1.1 million ha. These are significantly larger areas than the estimated 42,000 ha required for the current consumption of concentrates (average 1% of the dry matter intake). Concentrate production could possibly be done within existing agricultural land by increasing current yields. However, if this land demand would cause deforestation of secondary forests in Kenya, this would lead to C emissions of approximately 57 ± 2 Mt CO_2_eq for the medium intensification and of 118 ± 4 Mt CO_2_eq for the high intensification scenario. To put this additional land demand into context, at present, croplands and grazing lands occupy approximately 5 million ha of the dairy area, and the C emissions from the conversion of 1.1 million ha would be more than double the annual emissions (32 Mt CO_2_eq) of the Kenyan agricultural sector (Government of Kenya, 2015b).

#### 3.2.3 Forest C change

Forest C loss due to grazing was reduced in all scenarios by 374.4–2,113.3 Mg CO_2_eq/year (±98.0–493.3) relative to the baseline (Table S6). The scenarios producing maize silage with actual maize yields (FeCo–Ya: 374.4 ± 401.7 Mg CO_2_eq/year and FoFeCo‐Ya: 730.7 ± 493.3 Mg CO_2_eq/year) showed the lowest reduction of 48%–93% for forest C loss due to grazing. Realizing the water‐limited yield potential Yw at 50% and 80% lowered forest C loss due to grazing by two‐ to threefold (FoFeCo–Yw50%: 789.5 ± 78.9 Mg CO_2_eq/year; FeCo–Yw80%: 1,762.1 ± 392.5 Mg CO_2_eq/year), the land deficits were reduced and the production of maize on agricultural land in the vicinity of forests increased throughout the study area. Hence, closing the yield gap of maize could increase the forest C sink. The smallest deficit of arable land, the lowest forest C loss due to grazing and, therefore, the highest forest C sink potential were calculated for the FoCo scenario. This scenario showed the highest mitigation potential for agricultural land and for forests due to the highest reduction of GHG emission intensities by 33% and forest C loss by 270% while increasing milk production by 45% (Figure 6).

#### 3.2.4 GHG emission intensities

The GHG emission intensity of the baseline was 2.36 ± 0.05 kg CO_2_eq kg fat and protein corrected milk (FPCM); Figure 5b; Table 1). For the baseline and the scenarios emission sources included those from enteric fermentation, manure and soil management (cultivation of feed crops), fertilizer application, concentrate and C emissions from LUC. Most scenarios reduced GHG emission intensities. The only exception was the FoFeCo scenario, with actual maize yield (Yo) which increased GHG emission intensity by about 11% (2.64 ± 0.10 kg CO_2_eq/kg FPCM). Realizing Yw at 50% and 80% reduced emission intensity by 1%–17% compared to the baseline. The lowest emission intensity was shown for the FoCo scenario (1.68 ± 0.05 kg CO_2_eq/kg FPCM), with a reduction of 33%. Milk production increased in all scenarios by 44.0%–51.0% relative to the baseline.

**Table 1:** Dry matter intake, feed intake (per cow per year), milk yields (kg of fat corrected milk per cow per year), yield increase and GHG emission intensity for each livestock production system (LPS) and feeding scenario (Baseline, FeCo, FoFeCo and FoCo), including three levels of yield gaps for fodder maize (Yo: actual yields, Y50: closing gap at 50%, and Y80: closing gap at 80%)

LPS	Scenario	DMI (kg head^−1^ year^−1^)	Feed types (kg head^−1^ year^−1^)					Milk yield (kg FPCM head^−1^ year^−1^)	Milk yield increase (%)	GHG emission intensity (kg CO_2_eq/kg milk)
			Pasture	Napier grass	Maize stover	Maize silage	Concentrate			
MRA	Baseline									
	Yo	3,565	1,837	818	874	—	35	1,729	—	2.64
	FeCo									
	Yo	4,391	1,837	818	402	883	450	2,489	44.0	2.38
	Y‐50	4,391	1,837	818	402	883	450	2,489	44.0	2.04
	Y‐80	4,391	1,837	818	402	883	450	2,489	44.0	1.93
	FoFeCo									
	Yo	5,321	72	2,583	—	1,765	900	2,610	51.0	2.90
	Y‐50	5,321	72	2,583	—	1,765	900	2,610	51.0	2.45
	Y‐80	5,321	72	2,583	—	1,765	900	2,610	51.0	2.23
	FoCo									
	Yo	3,980	955	1,701	874	—	450	2,507	45.0	1.76
MRH	Baseline									
	Yo	3,621	1,233	1,321	1,031	—	36	1,881	—	2.40
	FeCo									
	Yo	4,454	1,233	1,321	554	896	450	2,708	44.0	2.16
	Y‐50	4,454	1,233	1,321	554	896	450	2,708	44.0	1.97
	Y‐80	4,454	1,233	1,321	554	896	450	2,708	44.0	1.94
	FoFeCo									
	Yo	5,883	—	3,113	77	1,793	900	2,840	51.0	2.64
	Y‐50	5,883	—	3,113	77	1,793	900	2,840	51.0	2.24
	Y‐80	5,883	—	3,113	77	1,793	900	2,840	51.0	2.20
	FoCo									
	Yo	4,035	337	2,217	1,031	—	450	2,727	45.0	1.71
MRT	Baseline									
	Yo	3,573	1,275	1,523	740	—	35	1,932	—	2.33
	FeCo									
	Yo	4,437	1,275	1,523	304	885	450	2,782	44.0	2.10
	Y‐50	4,437	1,275	1,523	304	885	450	2,782	44.0	1.94
	Y‐80	4,437	1,275	1,523	304	885	450	2,782	44.0	1.89
	FoFeCo									
	Yo	5,961	—	3,292	—	1,769	900	2,917	51.0	2.56
	Y‐50	5,961	—	3,292	—	1,769	900	2,917	51.0	2.19
	Y‐80	5,961	—	3,292	—	1,769	900	2,917	51.0	2.12
	FoCo									
	Yo	3,988	390	2,408	740	—	450	2,801	45.0	1.67
MIA	Baseline									
	Yo	3,565	1,837	818	874	—	35	1,736	—	2.62
	FeCo									
	Yo	4,391	1,837	818	402	883	450	2,500	44.0	2.36
	Y‐50	4,391	1,837	818	402	883	450	2,500	44.0	2.05
	Y‐80	4,391	1,837	818	402	883	450	2,500	44.0	1.93
	FoFeCo									
	Yo	5,321	72	2,583	—	1,765	900	2,621	51.0	2.88
	Y‐50	5,321	72	2,583	—	1,765	900	2,621	51.0	2.43
	Y‐80	5,321	72	2,583	—	1,765	900	2,621	51.0	2.24
	FoCo									
	Yo	3,980	955	1,701	874	—	450	2,517	45.0	1.77
MIH	Baseline									
	Yo	3,621	1,233	1,321	1,031	—	36	1,880	—	2.40
	FeCo									
	Yo	4,454	1,233	1,321	554	896	450	2,708	44.0	2.16
	Y‐50	4,454	1,233	1,321	554	896	450	2,708	44.0	1.98
	Y‐80	4,454	1,233	1,321	554	896	450	2,708	44.0	1.95
	FoFeCo									
	Yo	5,883	—	3,113	77	1,793	900	2,839	51.0	2.64
	Y‐50	5,883	—	3,113	77	1,793	900	2,839	51.0	2.28
	Y‐80	5,883	—	3,113	77	1,793	900	2,839	51.0	2.20
	FoCo									
	Yo	4,035	337	2,217	1,031	—	450	2,726	45.0	1.73
MIT	Baseline									
	Yo	3,573	1,275	1,523	740	—	35	1,931	—	2.34
	FeCo									
	Yo	4,437	1,275	1,523	304	885	450	2,781	44.0	2.11
	Y‐50	4,437	1,275	1,523	304	885	450	2,781	44.0	1.97
	Y‐80	4,437	1,275	1,523	304	885	450	2,781	44.0	1.92
	FoFeCo									
	Yo	5,961	—	3,292	—	1,769	900	2,916	51.0	2.57
	Y‐50	5,961	—	3,292	—	1,769	900	2,916	51.0	2.28
	Y‐80	5,961	—	3,292	—	1,769	900	2,916	51.0	2.25
	FoCo									
	Yo	3,988	390	2,408	740	—	450	2,801	45.0	1.69

## 4 DISCUSSION

### 4.1 Intensification of smallholder dairy farms and forest C change

In this study, cattle numbers on smallholder farms in Kenya's dairy production region were positively correlated to the loss of forest C, and to the presence of cattle grazing in adjacent forests. This is the first study that provides such a quantitative measure of the impact of cattle production on tropical forests at sectoral scale in East Africa. Hosonuma et al. (2012) reported that 8%–12% of the forest disturbance across SSA can be attributed to livestock grazing in forests. The empirical study by Brandt, Hamunyela, et al. (2018) found that cattle grazing across a tropical montane forest in Kenya was associated with forest disturbance, with evidence found on 75% of the sites visited during a recent forest survey. Furthermore, this study reported an increased risk of forest disturbance by up to 5% due to higher numbers of total cattle on larger farms located adjacent to the forest. The use of montane forests by smallholder farmers to graze livestock was also reported for Ethiopia (Baudron, Duriaux Chavarría, Remans, Yang, & Sunderland, 2017; Duriaux Chavarría, Baudron, & Sunderland, 2018), who found positive effects on dietary diversity and nutrient balances on farms located in the vicinity of the forests (distance = 5.5 km) used for grazing. The authors argue that the amount of herbaceous biomass removed from the forest through grazing is likely lower than the regrowth rates, although they did not quantify the impact of forest grazing on C loss. The results of our study show a net forest C loss due to dairy cattle within the neighbourhoods of nonintensified smallholder farms ranging in average between 566 and 842 kg C ha^−1^ year^−1^. Although these C losses are small, cattle grazing prevents forest regeneration, which affects the long‐term forest C sink.

In addition, our study found that improved cattle feeding and intensified milk production on smallholder farms were associated with lower forest C loss due to cattle grazing. Intensification of smallholder agriculture is postulated to reduce the pressure on forest ecosystems, because farm productivity could reduce the demand for land (Campbell, Thornton, Zougmoré, Asten, & Lipper, 2014). This process of intensification based on more nutritious feeds and improved dairy cattle moving away from extensive systems would reduce the negative impact on local, natural ecosystems such as forests (Wollenberg et al., 2011). Vallin et al. (2013) showed that intensification of livestock production could lower GHG emissions from deforestation in SSA, by improving management practices such as better quality feeds. Similarly, Caviglia‐Harris (2018) reported that the intensification of dairy production in Brazil could reduce the pressure on forests through pasture intensification. In Kenya, Brandt, Hamunyela, et al. (2018) quantified a 7% lower risk of forest disturbance when farms adjacent to forests had improved dairy cattle, attained higher milk yields and fed higher‐quality feeds. Thus, this study adds an important contribution to the current quantification of agricultural mitigation potentials providing empirical evidence of the connection between intensification of dairy and impacts on forest, and showing how to mitigate GHG emissions from forests.

### 4.2 Mitigation across the land use sector

The increase of livestock productivity through higher feed quality supports agricultural mitigation mainly by increasing feed conversion efficiency and lowering CH_4_ emissions from enteric fermentation (Herrero et al., 2016; Hristov et al., 2013; Knapp, Laur, Vadas, Weiss, & Tricarico, 2014). For instance, in Costa Rica, Wattiaux, Iñamagua‐Uyaguari, Casasola‐Coto, Guerra‐Alarcón, and Jenet (2016) showed the efficacy of improving feed quality in mitigating GHG emissions in intensified dairy farms. Similarly, our results of different feeding scenarios show potential to reduce the GHG emission intensity of dairy in East Africa. We present evidence that closing the yield gaps of maize could play a role in limiting CO_2_ emissions from LUC by reducing land demand and forest emissions due to grazing, which was previously argued using results from global modelling studies (Herrero et al., 2016; Valin et al., 2013; Weindl et al., 2017). In our study, the effect of avoided forest grazing on the reduction of emission intensity from forest C change range from 0.02 to 0.06 CO_2_eq/kg milk, which is small compared to the reduction of emission intensities from agricultural land alone. However, as demand for livestock products increase in Africa and the dairy sector intensifies further, competing demands for land are likely to create more trade‐offs between climate change mitigation strategies. Therefore, the approach presented here is a novel contribution to quantify the effect of intensification of feeding practices in dairy farms and at landscape level, including forests as an important C sink.

The increased N_2_O emissions due to higher fertilizer N application rates to close the yield gap of maize can be offset by reduced CO_2_ emissions from LUC on agricultural land due to lower land requirements as shown in this study (cf. Figure 5a), and modelled at coarser continental level (Havlík et al., 2014; Valin et al., 2013). The potential to close the yield gap of maize in Kenya is high (van Ittersum et al., 2013); maize is a staple crop in East Africa which is widely used to feed livestock as crop residue (Valbuena et al., 2012). However, the production of maize throughout the whole region does not meet the demand due to very low input use and poor productivity with average yields lower than 2 t/ha (van Ittersum et al., 2016). Therefore, Kenya relies on imported maize for human consumption importing over 700,000 tonnes each year (USDA, 2017). Consequently, increasing maize yields could contribute directly to food security and to the intensification of the livestock sector as shown in this study. Given the competing demands for land, intensification will increase the feasibility to implement feed improvement strategies, especially in regions with high human population and livestock densities (Brandt, Herold, et al., 2018; Gerssen‐Gondelach et al., 2017). Smallholder farmers across the Kenyan highlands often lack the land required to grow sufficient feeds of high quality (Bebe, 2008), which leads to off‐farm grazing on common lands such as forests. This study shows that improvements of quality and productivity of feed crops such as the African Napier grass can reduce GHG emission intensities and emissions from agricultural land, and reduce the loss of C from forests grazing.

Producing maize silage, such as in the FeCo and FoFeCo scenarios, would lead to reductions of forest C loss due to forest degradation, especially when yield gaps are closed. Realizing the water‐limited yield potential for maize at least at 50%, could turn forests into C sinks as the C gain exceeds the C loss due to cattle grazing. Increasing livestock productivity could result in land sparing as indicated in the global studies by Havlík et al. (2014), Kreidenweis et al. (2018) and Valin et al. (2013). This study adds empirical evidence to the results of these coarse modelling exercises by including spatial relationships between local farm practices, land availability and forest C change. The characteristics of these relationships might be context‐specific and determined by market and infrastructure development, and therefore should be included into assessments of climate change mitigation that aim to determine the potentials for sustainable intensification at sectoral level.

Land requirement and the deficit of arable land would be lowest for the FoCo scenario, which improves forage quality by cultivating Napier grass and adding more concentrate to the diet. Under this scenario, there would be much less forest grazing to meet feed deficits on agricultural land, and the forest C loss would be reduced almost threefold, showing the largest forest C sink potential among scenarios. However, the amount of concentrates needed for all scenarios (Tables 2 and 3), could cause indirect LUC increasing absolute GHG emissions because of the low crop yields. With appropriate policies to limit LUC, the FoCo scenario could result in a net benefit for Agriculture, Forest and Other Land Uses (AFOLU) mitigation by reducing GHG emissions across the agricultural and forest sectors effectively, since total agricultural GHG emissions and forest C change combined would be 2.5% lower than in the baseline. The current national dairy master plan and the dairy NAMA seek feed options that realize milk yield gains and mitigation benefits (Government of Kenya, 2010, 2017). The feed intensification strategies combined in the FoCo scenario represent promising technical ‘win‐win’ options for the dairy sector.

**Table 2:** Amount of concentrate and cow population for each livestock production system (LPS) of the study area under the medium (FoCo and FeCo) and high intensification (FoFeCo) scenarios

LPS	Population dairy cows (*n*)	Productive dairy cows (*n*)	Concentrate use		
			Baseline (tonnes)	FoCo and FeCo (tonnes)	FoFeCo (tonnes)
MRA	246,237	147,742	16,965	66,484	132,968
MRH	111,205	66,723	2,513	30,025	60,051
MRT	2,443,342	1,466,005	16,965	659,702	1,319,405
MIA	28,845	17,307	16,965	7,788	15,576
MIH	20,376	12,225	3,142	5,501	11,003
MIT	142,081	85,249	6,283	38,362	76,724
Total	2,992,086	1,795,251	62,834	807,863	1,615,726

Abbreviations: MIA, mixed irrigated system in arid areas; MIH, mixed irrigated system in humid areas, MIT, mixed irrigated system in tropical highlands; MRA, mixed rainfed system in arid areas; MRH, mixed rainfed system in humid areas; MRT, mixed rainfed system in tropical highlands.

**Table 3:** Calculation of land footprint (ha) as the amount of land to produce the concentrates for dairy cows under the baseline and medium (FoCo and FeCo) and high intensification scenarios (FoFeCo). The land footprint accounts for the composition of the concentrates, with the ingredients from Weiler et al. (2014)

Ingredients^a^	Yield^b^	Area	Baseline		FoCo and FeCo		FoFeCo	
	t/ha	ha	Tonnes	ha	Tonnes	ha^c^	Tonnes	ha^c^
Rice bran	2.68	97,659	16,965	6,330	218,123	75,059	436,246	156,448
Lime	10.78	1,693	2,513	233	32,315	2,764	64,629	5,762
Wheat grain	1.93	85,732	16,965	8,790	218,123	104,227	436,246	217,244
Maize	1.52	2,092,459	16,965	11,161	218,123	132,341	436,246	275,843
Sunflower cake	0.97	11,840	3,142	3,239	40,393	38,404	80,786	80,046
Cotton seed cake	0.50	25,980	6,283	12,567	80,786	149,006	161,573	310,578
Total		2,315,363	62,834	42,321	807,863	501,800	1,615,726	1,045,921

a Composition of concentrates includes 27% rice bran, 4% lime, 27% wheat, 27% maize, 5% sunflower cake and 10% cotton seed cake.

b Yields and areas harvested for the whole Kenya from FAOStat (2019). Rice bran imported from Uganda; the rest of the ingredients produced in Kenya.

c Area calculated as the area required to produce the tonnage of ingredients, minus the area allocated in the baseline.

### 4.3 Policy relevance: Targeting and financing the implementation of CSA practices

This study adds value to current policy debates in SSA on the contribution of the agriculture and forestry land use sectors to climate change mitigation by quantifying the intersection between smallholder intensification in the Kenyan dairy sector and the reduction of emissions from forests. This approach evaluates the effectiveness of CSA practices to mitigate AFOLU emissions in the context of developing agricultural production at sectoral level. Several multiobjective modelling tools have been developed to support decision‐making processes, which aim to prioritize CSA practices based on evidence by integrating qualitative and quantitative information at various spatial and temporal scales (e.g. Brandt, Kvakić, Butterbach‐Bahl, & Rufino, 2017; Dunnet et al., 2018). This study provides critical empirical data for such tools to explore the feasibility of CSA practices by considering land availability and how to mitigate GHG emissions from agricultural land and forests.

Policy instruments such as NAMAs and NDCs aim to enable the development of climate‐smart food production and must, therefore, rely on compelling evidence that shows the potential of ‘win‐win’ solutions to benefit smallholder farmers and to contribute to climate mitigation goals (Grassi et al., 2017; Lipper et al., 2014). In addition, mitigation policies need to support the creation of economic incentives to foster the implementation of CSA practices and to reduce adoption barriers (Lipper et al., 2014). However, local and national policies need to incentivize development without causing rebound effects that can offset any gains in GHG emissions at farm or regional scales. Such offsets may be driven by feedbacks between improved farm practices and market responses triggering regional expansion of dairy farming. Effective policies have to incorporate mechanisms to avoid negative effects on forests and climate. Climate financing schemes require quantitative information on productivity gains and mitigation potentials of specific practices to inform decisions on investments targeted at farm level with positive impact at landscape level (Reed, Vianen, Deakin, Barlow, & Sunderland, 2016). Agricultural practices may affect tropical forests and their C dynamics by removing biomass through cattle grazing, which prevents forest regeneration (Hosonuma et al., 2012; Pearson et al., 2017). The added value of this analysis is that it integrates direct effects of farm intensification on agricultural land and the indirect effects on forest use and emissions. These results highlight the need for policymakers in agricultural and forests sectors to work together and to develop more integrated policy frameworks based on the CSA concept and policies on ‘Reducing emissions from deforestation and forest degradation’ (REDD+) as discussed by Carter, Arts, et al. (2018) and Carter, Herold, et al. (2018).

### 4.4 Limitations of this study and future research

To our knowledge, this is the first assessment that quantifies the local impact of livestock on forest C change by linking spatially explicit data, dynamic livestock modelling and farm surveys to remote‐sensing data for the region. The relationship between farming practices and forest C loss (at a distance of 5 km) was determined empirically (Brandt, Herold, et al., 2018) and should be taken with caution when extrapolating to other regions. This distance may depend on the region‐specific land use dynamics. Thus, more research is necessary to characterize local interactions between farms and forests using information on local farming practices and landscape configurations. Measurements obtained from grazing experiments for different forest and livestock types are required to estimate the direct impact of cattle on above‐ and below‐ground carbon stocks in forests (Schulz et al., 2016) and resulting GHG emissions. Livestock movement patterns can be traced through telemetry analyses to gain knowledge about distances that cattle walk and the time they spend inside forests (Gao, Kupfer, Zhu, & Guo, 2016). Aggregated spatially and temporally, this ground information could be used to calibrate and validate the estimates of forest C change related to livestock grazing derived from remote‐sensing data.

The increases of maize yields in this study were based on increased application rates of synthetic N fertilizer. Realizing the water‐limited yield potential of maize at 80% requires high N inputs of 69–185 kg N/ha (ten Berge et al., 2019; van Bussel et al., 2015). Fertilizer, transport and labour costs of high N application rates, however, may render the intensification of feed production economically unfeasible or simply unattractive for smallholder farmers, if economic returns from milk sales do not justify these investments. Consequently, moderate application rates of synthetic fertilizer of 60–90 kg N/ha could be more realistic from a farmer's point of view in the Kenyan highlands (Mucheru‐Muna, Mugendi, Kung'u, Mugwe, & Bationo, 2007; Mucheru‐Muna et al., 2014). Therefore, reliable market prices for milk and improved access to markets are required for smallholders to adopt practices, which aim at closing the yield gap of crops such as maize. To increase the adoption of practices that improve the quality of dairy feeds, assessments of agricultural productivity and climate change mitigation have to be coupled with cost‐benefit analyses that take into account seasonal variation of costs and returns and farm distance to markets to find optimal cost‐benefit ratios for smallholder farmers (Kibiego, Lagat, & Bebe, 2015). Moreover, apart from abiotic and biotic factors such as climate, soils and cultivar traits, crop management practices determine the potential to improve maize yields (Kiboi et al., 2017; Rattalino Edreira et al., 2018). Hence, the dissemination of tailored knowledge about best practices in a certain farming context through agricultural extension or web and mobile‐based information services are crucial.

Greater efforts to intensify smallholder agriculture sustainably have to be undertaken by agricultural development programmes to improve crop and livestock yields and to achieve food security in SSA (van Ittersum et al., 2016). More food produced from existing agricultural land will be required to feed the continent's fast growing human population. Considering the shrinking of farm sizes and the increasing shortage of arable land in SSA (Vanlauwe et al., 2014), research at landscape level has to be strengthened to explore the boundaries within which smallholder agriculture can be intensified sustainably to safeguard food security. This study estimated high emissions from C leakage effects that could result from the displacement of GHG emissions due to the increased demand for dairy concentrates, which could trigger cropland expansion into natural ecosystems outside the dairy production area as indicated by other studies (Styles, Gonzalez‐Mejia, Moorby, Foskolos, & Gibbons, 2018). To account for the increased demand of land, we calculated the land footprint due to the amount of concentrate required across scenarios. This measure is only an indication of the displacement of C emissions that could arise from the feed intensification explored here. The estimated land requirement indicates a 10%–20% increase in the amount of land (0.5–1.1 million ha) currently dedicated to feed the dairy cows in the study area, which would be a risk for C leakage. We estimate that cropland expansion into Kenyan forests to produce the ingredients for concentrates could produce GHG emissions equivalent to two‐ to fourfold the annual emissions from the whole agricultural sector. Because forest policies are likely to become more stringent in the future, it is more likely that the demand of concentrates is met by intensifying the use of croplands and grasslands.

To properly capture off‐farm C emissions there is a need for a detailed mapping of the concentrate value chain and a consequential life cycle assessment as applied in the case study of Styles et al. (2018). The market‐oriented stimulation of the agricultural sector could lead to rebound effects due to reduced production costs, higher demand and, therefore, increasing production (Kreidenweis et al., 2018; Valin et al., 2013). A higher demand for grain‐based concentrates may spark the land use competition to cultivate livestock feeds versus food, especially without closing yield gaps. Thus, analyses of AFOLU mitigation need to integrate effects along supply chains, and aggregate effects from farm, and landscapes to the sectoral level. Such assessments should be coupled with economic models to provide estimates of effective mitigation potentials by incorporating feedbacks between markets and agricultural development.

## 5 CONCLUSIONS

Improving the quality of dairy feeds can have climate change mitigation benefits for agriculture and forests and can contribute to food security by increasing milk yields in Kenya. Closing the yield gap of maize could increase the adoption of better feeding practices, and can reduce GHG emission intensities from milk production and the loss of C in local forests. The largest mitigation benefits across land use sectors could be achieved by improving forage quality by feeding more African Napier grass to cows and supplementating concentrates. There might be additional risks of LUC associated with the production of concentrates that deserve to be studied in more detail. These findings emphasize the importance of assessing the impact of specific CSA practices prior to their recommendation for climate mitigation programmes. Targeting and prioritization at high spatial resolution to identify mitigation potentials across land use sectors can reveal implementation constraints such as land availability. Top–down assessments conducted at coarse continental scales do not capture local and landscape level contexts, which may render the implementation of targeted interventions unfeasible or may reduce the effectiveness of mitigation outcomes. Integrated mitigation and development policy frameworks and climate financing instruments could benefit from the approach presented here to prioritize the most effective CSA practices and to invest into options that show the most promising potentials for sectoral development and climate change mitigation.
