# Supplementary material for: Intensification of dairy production can increase the GHG mitigation potential of the land use sector in East Africa
Source: Glob Chang Biol. 2019 Nov 19;26(2):568–85. doi: 10.1111/gcb.14870 (PMC7027483; doi:10.1111/gcb.14870)
Supplement: Supplementary file 1 [file GCB-26-568-s001.docx]

**Supplementary material: Intensification of dairy production can increase the GHG mitigation potential of the land use sector in East Africa**

**1. Calculation of milk yields and GHG emissions from smallholder dairy production**

**1.1 Livestock simulator: LivSim**

The livestock production model LivSim was used to compute milk yields and GHG emissions from dairy cattle (exotic *Bos Taurus* and crosses between *Bos taurus* and local zebu *Bos indicus*), typically found on smallholder dairy farms in Kenya (Rufino et al., 2009). LivSim is a dynamic production model that simulates growth, milk yields, and faecal and urine excretion for individual dairy cattle on a monthly basis taking into account the animal’s energy and protein growth, maintenance and production requirements following AFRC (1993). Because LivSim is designed to simulate the impact of farmer’s resource allocation on lifetime productivity and output of cattle several parameters are required for running the model (Table S1). For instance, intake is dependent on feed quality and body size, therefore, mature weight and milk ME parameters are inputs to the model. Other parameters include the calving rates and dry period to calculate the reproduction potential of dairy cows. Productivity was assessed by lactation length, milk production, weight gain and manure production. Despite LivSim’s capability to calculate involuntary culling this functionality was not used under this framework because it is assumed that improved feeds used in all scenarios will prevent death from malnutrition within one year. The animal manure module HeapSim, which is integrated into LivSim simulates the dynamics of nutrients, including losses depending on the collection, storage and use of manure (Rufino et al, 2007).

Table S1: LivSim Model input and parameters adapted from Rufino et al (2009).

| Parameters | Parameter values | Units |
| --- | --- | --- |
| Mature weight | 500 | kg |
| Calf birth weight | 30 | kg |
| Weaning age | 3 | month |
| Minimum calving rate (poor condition) | 0.25 | y^-1^ |
| Maximum calving rate (good condition) | 0.90 | y^-1^ |
| Pregnancy length | 298 | d |
| Postpartum length | 2 | month |
| Milk fat (average) | 34 | g kg^-1^ |
| Milk crude protein (average) | 32.0 | g kg^-1^ |
| Milk metabolisable energy (average) | 19.4 | MJ (kg DM)^-1^ |
| Dry period | 2 | month |
| Maximum milk yield | 4,450 | kg lactation^-1^ |
| Daily average of maximum milk yield | 14.6 | kg d^-1^ |
| Lactation length | 10 | month |

**1.2 GHG emission sources**

The GHG emission module of LivSim computes the emissions from smallholder dairy production during the simulations, including: CH_4_ emissions from enteric fermentation, CH_4_ emissions from manure management, direct and indirect N_2_O emissions from manure management, direct and indirect N_2_O emissions from managed soils, and N_2_O and carbon dioxide (CO_2_) emissions from LUC resulting from the conversion of grazing land to cropland used for the cultivation of maize for silage and Napier grass (*Pennisetum purpureum*) (Brandt et al., 2018). The approach to calculate GHG emissions and emission parameters follows the tier 2 methodology of IPCC (2006), including additional data appropriate for the regional context of the analysis (Table S2). A classification of livestock production systems (LPS, Robinson et al., 2011) was used to capture differences in agro-climatic conditions prevalent across the dairy region in Kenya, which translate into variations of feed quality and productivity, and lead to differences in milk yields and GHG emissions. Computed CH_4_ and N_2_O emissions were converted into CO_2_eq, based on global warming potentials from the 5^th^ assessment report of the ‘Intergovernmental Panel on Climate Change’ (IPCC, 2014). GHG emissions were calculated as total GHG emissions and emission intensities. Intensities are reported on product (fresh milk) basis and converted into kg CO_2_eq per kg fat and protein corrected milk (FPCM).

Table S2: Descriptions of parameters, values and uncertainty ranges (min, max), used to compute CH_4_, N_2_O and CO_2_ emissions from enteric fermentation (EF), manure management (MM), managed soils (MS), including land use changes, and dairy concentrate supplementation. Abbreviations of livestock production systems (LPS) are: mixed rainfed system in arid areas (MRA), mixed rainfed system in humid areas (MRH), mixed rainfed system in tropical highlands (MRT), mixed irrigated system in arid areas (MIA), mixed irrigated system in humid areas (MIH) and the mixed irrigated system in tropical highlands (MIT). This table was adapted from SI Table S1 in Brandt et al. (2018).

| Parameter | Equation | Value / range | Reference |
| --- | --- | --- | --- |
| Net energy maintenance (EF) | Eq. 10.3 | Cf = 0.386 for lactating cow  Cf = 0.322 for non- lactating cow | IPCC (2006, table 10.4) |
| Net energy activity (EF) | Eq. 10.4 | Ca = 0.17 for grazing  Ca = 0 for non-grazing | IPCC (2006, table 10.5) |
| Net energy growth (EF) | Eq. 10.6 | BW = calculated by LivSim  C = 1  WG = calculated by LivSim | IPCC (2006, equation 10.6), Rufino et al. (2009) |
| Net energy lactation (EF) | Eq. 10.8 | Milk yield = calculated by LivSim  Fat (%) = calculated by LivSim (2.7 – 4.2)  Lactation state = calculated by LivSim | Rufino et al. (2009) |
| Net energy pregnancy (EF) | Eq. 10.13 | Cpregnancy = 0.1  Pregnancy state = calculated by LivSim | IPCC (2006, table 10.7), Rufino et al. (2009) |
| Ratio net energy available for maintenance to digestible energy consumed (EF, MM) | Eq. 10.14 | DE = feed type specific (%):   - Grazing land: 45 – 55 - Napier grass: 50 – 60 - Maize stover: 45 – 55 - Maize silage: 50 – 60 - Dairy concentrate: 75 – 85 | IPCC (2006), Anindo et al. (1994), Rufino et al. (2009) |
| Ratio net energy available for growth to digestible energy consumed (EF, MM) | Eq. 10.15 | DE = see above | IPCC (2006) |
| Gross Energy (EF, MM) | Eq. 10.16 | DE = see above | IPCC (2006) |
| CH_4_ emissions (EF) | Eq. 10.21 | Ym (%) = 5.5 – 7.5 | IPCC (2006, table 10.12) |
| Volatile solid excretion (MM) | Eq. 10.24 | DE = see above  UE*GE = 0.04  ASH = 0.08 | IPCC (2006, equation 10.24) |
| CH_4_ emissions (MM) | Eq. 10.23 | Bo(T) = 0.11 – 0.15  MCF storage (%) = 1.5 – 2 (dry lot) and 4 -5 (solid storage)  MCF grazing (%) = 1.5 - 2  MCF application (%) = 0.5 - 1  MS = proportion of manure stored based on ratio between grazing land (not managed) and cropland used for feed production (managed) | IPCC (2006, table 10.17 and 10A-4) |
| N excretion (MM) |  | N content of urine and faeces = calculated by LivSim | Rufino et al. (2009) |
| Direct N_2_O emissions (MM) | Eq. 10.25 | EF3 (kg N_2_O–N) = 0.01 – 0.02 (dry lot) and 0.0025 – 0.0075 (solid storage) | IPCC (2006, table 10.21), Herrero et al. (2013) |
| N volatilization as NH_3_ and NO_x_ (MM) | Eq. 10.26 | MS = see above  FracGasMS (%) = 30 – 50 (dry lot), 30 – 40 (solid storage) | IPCC (2006, table 10.22), Herrero et al. (2013) |
| N losses due to leaching (MM) | Eq. 10.28 | MS = see above  FracleachMS (%) = 0 – 20 (dry lot) and 5 – 15 (solid storage) | IPCC (2006, table 10.23), Herrero et al. (2013) |
| Indirect N_2_O emissions (MM) | Eq. 10.27 and 10.29 | EF4 (kg N_2_O–N) = 0.02 – 0.01  EF5 (kg N_2_O–N) = 0.0005 - 0.0075  N_2_O = N * 44 / 28 | IPCC (2006) table 11.3), Herrero et al. (2013) |
| N from animal manure applied to soils used for feed production (MS) | Eq. 11.4 | FracFEED = 1 - MSO (%) (fraction of manure for other uses) 0.1 - 0.2 | IPCC (2006), Herrero et al. (2013) |
| N in urine and dung deposited on pasture (MS) | Eq. 11.5 | 1 – MS = fraction of manure dropped on grazing land | IPCC (2006) |
| Direct N_2_O emissions from manure deposited on pastures (MS) | Eq. 11.1 | EF3PRP (kg N_2_O–N) = 0.007 – 0.06 | IPCC (2006) table 11.3), Herrero et al. (2013), Rosenstock et al. (2016) |
| N in synthetic fertilizer (MS) | Eq. 11.1 | FSN (kg ha^-1^) = spatially explicit dataset | Potter et al. (2010) |
| N in crop residues (above and below ground) (MS) | Eq. 11.7 and 11.7A | Fcr = calculated per crop (Napier grass and maize)  Yields (kg ha^-1^):  Napier grass = 12,000 (MRA, MIA) – 16,000 (MRH, MIH)  Maize = 2,335 (MRA, MIA) – 4,747 (MRH, MIH)  FracRemove = 0.75  Area burnt = 0  Other values taken from table 11.2, for Napier grass = perennial grasses | IPCC (2006, table 11.2),  Monfreda et al. (2008), Katiku et al. (2011), Weiler et al. (2014), Castellanos-Navarrete et al. (2015) |
| Mineralised N in mineral soils due to loss of soil carbon through land use change | Eq. 11.8 | R (C:N ratio) = spatially explicit dataset  Area = calculated, see reference | Hengl et al. (2015),  Brandt et al. (2018) |
| Direct N_2_O emissions (MS) | Eq. 11.1 | EF1 (kg N_2_O–N) = 0.003 – 0.005 (MRA, MIA) and 0.003 – 0.02 (MRH, MIH) and 0.003 – 0.01 (MRT) and 0.0004 – 0.036 (MIT) | IPCC (2006, table 11.1 and 11.2), Herrero et al. (2013), Hickmann et al. (2017) |
| N_2_O-N from atmospheric deposition (MS) | Eq. 11.9 | FracGASM (kg N) = 0.05 – 0.3 (dry lot) and 0.05 – 0.2 (solid storage)  FacGASF (kg N) = 0.03 – 0.3  EF4 (kg N_2_O–N) = 0.002 – 0.01 | IPCC (2006, table 11.3), Herrero et al. (2013) |
| N_2_O-N from leaching/runoff (MS) | Eq. 11.10 | FracLEACH-H (kg N) = 0.1 – 0.3  EF5 (kg N_2_O–N) = 0.005 - 0.0075 | IPCC (2006, table 11.3), Herrero et al. (2013) |
| Indirect N_2_O emissions (MS) | Eq. 11.9 and 11.10 | Fcr = calculated per crop (Napier grass and fodder maize)  N_2_O = N_2_O –N * 44 / 28 | IPCC (2006, table 11.2 and 11.3), Herrero et al. (2013) |
| CO_2_ emissions from land use change (grazing land to cropland) |  | Soil carbon loss (kg C ha^-1^ yr^-1^) = 11.1 – 688.2  Area = calculated, see reference  CO_2_ = Soil carbon loss * 44 / 12 | Don et al. (2011),  Brandt et al. (2018) |
| CO_2_eq emissions from the production of dairy concentrates |  | Emission factor (kg CO_2_eq kg concentrate^-1^) = 1.36 | Weiler et al. (2014) |
| Cattle densities |  | Spatially explicit dataset (heads km^-2^) used to upscale GHG emissions and milk production.  Grid cell uncertainty ranges are calculated based on:  RMSE of 0.42 = log (x + 1) | Robinson et al. (2014) |

**2. Upscaling Approach**

Output tables of GHG emissions and milk yields generated by LivSim were upscaled and mapped by using spatially explicit data on LPS and cattle density at a spatial resolution of 1x1 km (Fig. S1, Robinson et al., 2011, 2014). Based on cattle density and sub-national, county-level data on cattle types, the density of dairy cattle was calculated (Government of Kenya, 2014). Herd composition data were used to estimate the proportion of productive and non-productive animals in dairy Kenyan herds (Bebe et al., 2002).


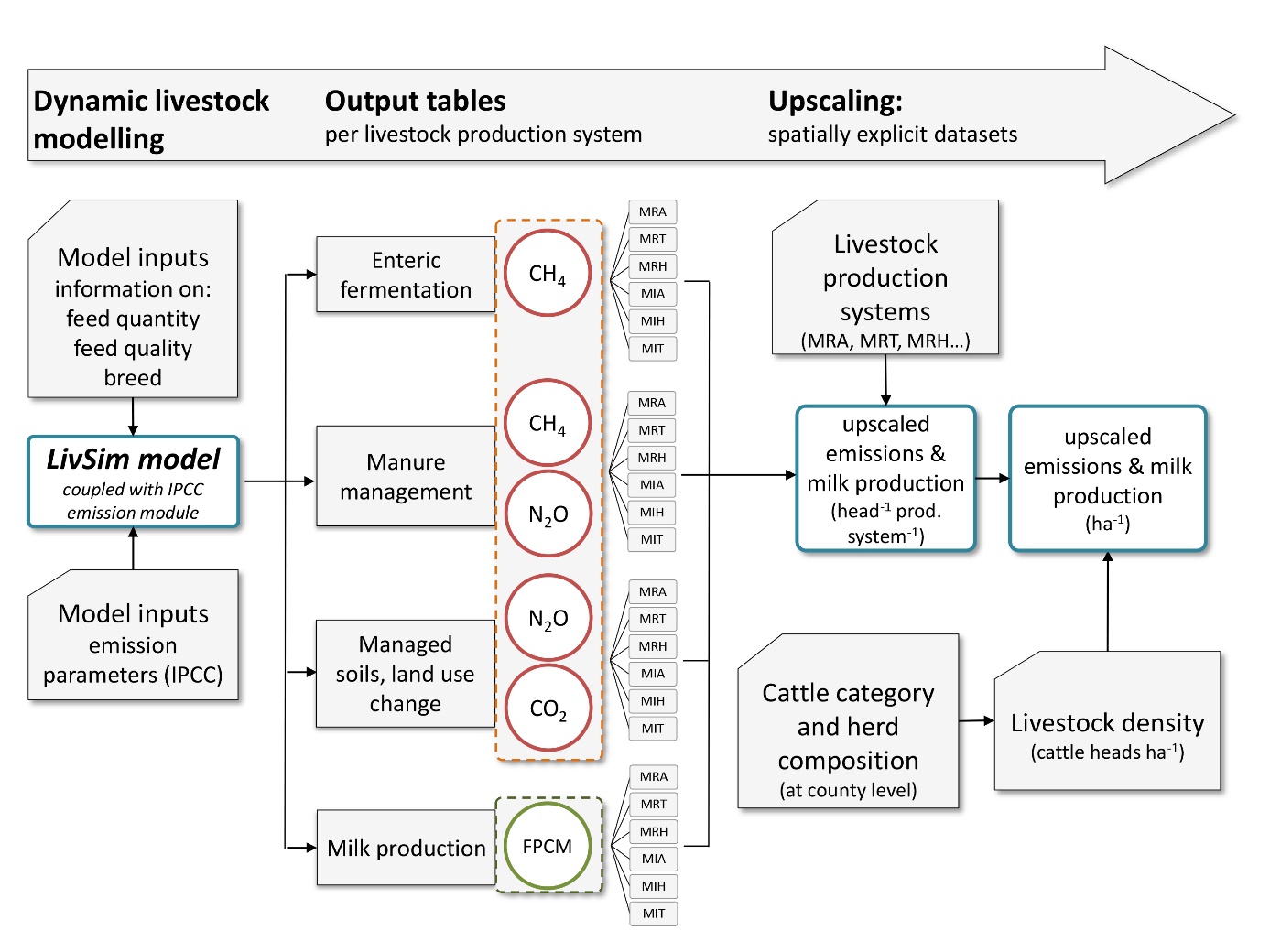


Fig. S1: Flowchart of the model framework applied consisting of the dynamic livestock model LivSim (Rufino et al., 2009) to compute output tables of GHG emissions and milk production (FPCM = fat and protein corrected milk). Subsequently, these tables were spatially up-scaled based on livestock production systems (LPS) and mapped using data on cattle herd composition, cattle category and density. This figure is adapted from Brandt et al. (2018).

**3. Feed improvements: baseline and scenarios**

**3.1 Baseline feeds**

Feed composition and quantities used as model inputs in LIVSIM correspond to typical diets for smallholder dairy across the Kenyan highlands. The ingredients included in the scenarios are commonly found in the dairy sector (Figure S2). Input data taken from literature and are reported in Table S6.


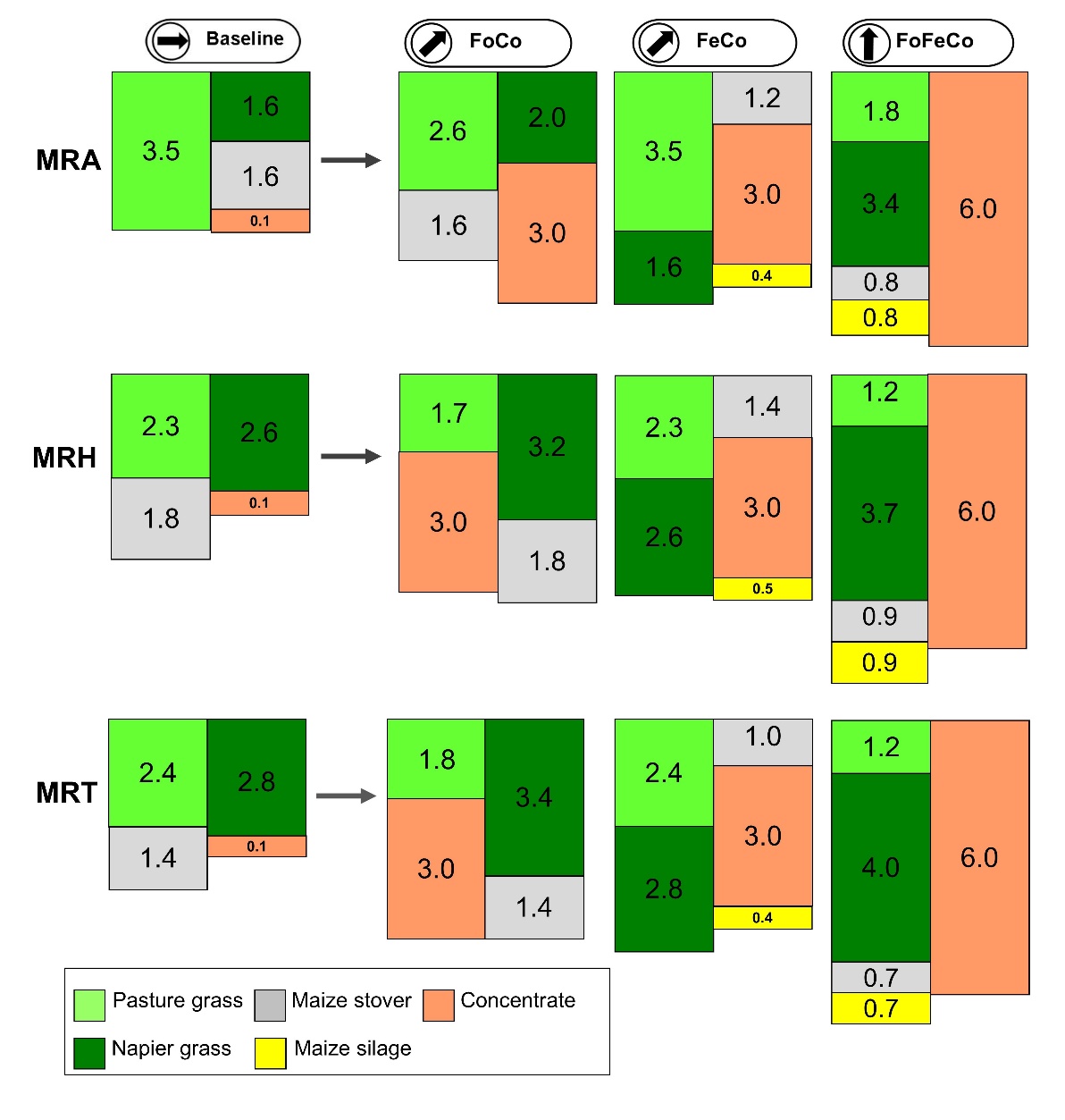


Figure S2: Proportional changes in feeds to baseline feeds in different livestock production systems namely: MRA, MRH and MRT measured in kg day^-1^ per tropical livestock unit (TLU). Feed changes in irrigated systems mirrored those in rainfed systems (i.e., MRA = MIA, MRH = MIH and MRT = MIT). Changes to feeds are shown for medium intensification scenarios (i.e., FeCo and FoCo) and high intensification scenario (FoFeCo). Figure adapted after Brandt et al., (2018).

**3.2 Feed improvement scenarios**

Three feed improvement scenarios were explored in this study consisting of combinations of three feed intensification strategies (Table S3). For the strategies of ‘improved forage quality’ (Fo), ‘feed conservation & increased grain content’ (Fe), low quality baseline feeds were replaced proportionally by higher quality feed alternatives at medium and high intensification levels relative to baseline dry matter intake (DMI). For the scenarios that contain the Fe strategy, water-limited yield potentials (Yw) of fodder maize were realized at 50% and 80%. For the strategy of ‘dairy concentrates supplementation’ (Co), supplementation rates were increased during the first 150 days of the lactation.

Table S3: Overview of feed improvement scenarios reflecting i) the composition of the three feed intensification strategies included, ii) the proportional changes of feed types referring to dry matter intake of the baseline feed and iii) the levels at which the water-limited yield potentials (Yw) for fodder maize are realised. Ya = actual maize yield, Yw-50 = water-limited yield potential realised at 50 %, Yw-80 = water-limited yield potential realised at 80 %.

| Feed intensification strategies | Intensification levels | Proportional change of feed types | Yield levels | Feed improvement scenarios | | |
| --- | --- | --- | --- | --- | --- | --- |
|  | | | | FeCo | FoCo | FoFeCo |
| Improved forage quality (Fo) | medium | + 25 % Napier grass  - 25 % pasture grass | Ya |  | **x** |  |
|  |  |  | Yw-50 |  |  |  |
|  |  |  | Yw-80 |  |  |  |
|  | high | + 50 % Napier grass  - 50 % pasture grass | Ya |  |  | **x** |
|  |  |  | Yw-50 |  |  | **x** |
|  |  |  | Yw-80 |  |  | **x** |
| Feed conservation & increased grain content (Fe) | medium | + 25 % maize silage  - 25 % maize stover | Ya | **x** |  |  |
|  |  |  | Yw-50 | **x** |  |  |
|  |  |  | Yw-80 | **x** |  |  |
|  | high | + 50 % maize silage  - 50 % maize stover | Ya |  |  | **x** |
|  |  |  | Yw-50 |  |  | **x** |
|  |  |  | Yw-80 |  |  | **x** |
| Dairy concentrates supplementation (Co) | medium | + 3 kg day^-1^ dairy meal | Ya | **x** | **x** |  |
|  |  |  | Yw-50 | **x** |  |  |
|  |  |  | Yw-80 | **x** |  |  |
|  | high | + 6 kg day^-1^ dairy meal | Ya |  |  | **x** |
|  |  |  | Yw-50 |  |  | **x** |
|  |  |  | Yw-80 |  |  | **x** |

**3.3 Closing yield gaps of fodder maize**

Actual maize yields (Ya) used in the baseline model, and Ya, water-limited yield potentials (Yw) and N requirements applied to feed improvement scenarios that contain the Fe (feed conservation & increased grain content) strategy are shown in Table S4. Data on Yw and N requirements were obtained from the ‘Global Yield Gap Atlas’ (GYGA).

Table S4: Actual yields (Ya), water-limited yield potentials (Yw) of fodder maize realised at 50 % and 80 % and related N requirements. Abbreviations of livestock production systems (LPS) are: mixed rainfed system in arid areas (MRA), mixed rainfed system in humid areas (MRH), mixed rainfed system in tropical highlands (MRT), mixed irrigated system in arid areas (MIA), mixed irrigated system in humid areas (MIH) and the mixed irrigated system in tropical highlands (MIT)

| LPS | Ya maize | Yw-50 | | | Yw-80 | | |
| --- | --- | --- | --- | --- | --- | --- | --- |
|  |  | Yw maize  (kg ha^-1^) | | N requirements  (kg N ha^-1^) | Yw maize  (kg ha^-1^) | | N requirements  (kg N ha^-1^) |
| MRA | 1,399 | 2,187 | 38 | | 3,500 | 69 | |
| MRH | 1,485 | 4,659 | 94 | | 7,454 | 170 | |
| MRT | 1,566 | 4,294 | 85 | | 6,871 | 155 | |
| MIA | 1,713 | 2,385 | 36 | | 3,816 | 65 | |
| MIH | 1,516 | 4,774 | 100 | | 7,638 | 183 | |
| MIT | 1,644 | 4,984 | 102 | | 7,974 | 185 | |

**4. Land requirements to feed dairy cattle and shortage of available land**

We calculated the requirement of cropland needed to cultivate Napier grass for the feed improvement scenarios that contain the ‘Forage quality’ (Fo) strategy and fodder maize in the feed improvement scenarios that contain silage (Fe) strategy. The requirement for cropland was estimated based on actual feed crop yields (Ya) and water-limited yield potentials (Yw) for fodder maize, crop-specific feed intake per cow, density of dairy cattle per grid cell (Table S4; SI equations 6-7). The deficit of arable land was estimated for grid cells where the demand for cropland exceeded the amount of available grazing land. To calculate land demand the following equation was used:

$Land demand \left( {ha TLU}^{-1} \right)=\sum_{i=1}^{n} \frac{{feed intake}_{\left[ crop i \right]}\left( {kg TLU}^{-1} \right)}{{crop yield}_{\left[ crop i \right]}\left( {kg ha}^{-1} \right)}$ (1)

$${Land availability (ha km}^{-2}) = {available grazing land (ha km}^{-2})- {Land demand (ha TLU}^{-1})\times{dairy cattle density (TLU km}^{-2})$$

The CO_2_ emissions from land use change including only conversion grazing land to cropland, were calculated using changes in soil organic carbon (SOC data) for tropical soils from Don et al. (2011) and Hengl et al (2015), and spatially-explicit dataset on the extent of grazing lands by Velthuizen et al. (2007). The data from Don et al. (2011) were derived from a comprehensive meta-analysis that collated land use conversions in tropical soils. The dataset does not differentiate crops grown in croplands and therefore we used the same average loss of 6 tonnes of C per ha for the conversion of grasslands to generic cropland and apply it to the conversion of grazing land to cultivate Napier grass and maize. In practice, these land use conversions may differ on the cumulative C losses caused. We estimated maximum and minimum values for annual C loss (Table S2), and these were used to calculated emissions for land conversion, expressed as CO_2_eq. We did not distinguish between conversion to Napier grass and maize cultivation. There are often differences between annual and pluri-annual crops in terms of C storage in the soil. However, our previous research in East Africa showed that fields that receive large amounts of organic fertilisers such as Napier grass fields, tend to have much larger non-CO_2_ GHG emissions than annual crops (Rosenstock et al 2016). So it is likely that the aggregated emissions of the conversion of these two crops are similar, and therefore we assumed the same emission factors.

Table S5: Data used to analyse land requirements and shortage of available land for feed improvement scenarios.

| Data | Type | Reference |
| --- | --- | --- |
| Maize yields | actual yields (Ya): literature and spatially explicit dataset with a grid cell resolution of 10x10 km (kg ha^-1^ yr^-1^);  mean: 879±130 kg ha^-1^  water-limited yield potentials (Yw): ‘Global Yield Gap Atlas’ (GYGA) | Castellanos-Navarrete et al. (2015), Monfreda et al. (2008), Weiler et al. (2014)  van Ittersum et al. (2013) |
| Napier yields | literature (kg ha^-1^ yr^-1^)  mean: 1221±325 kg ha^-1^ | Katiku et al. (2011), Weiler et al. (2014), Castellanos-Navarrete et al. (2015) |
| Feed intake | literature (kg TLU^-1^ yr^-1^)  reported in Table 3 | Rufino et al. (2009), Katiku et al. (2011), Herrero et al. (2013), Weiler et al. (2014), Castellanos-Navarrete et al. (2015) |
| Dairy cattle density | tabular data at county level (%), spatially explicit dataset with a grid cell resolution of 1 x 1 km (TLU km^-2^) | Government of Kenya (2014), Robinson et al. (2014) |
| Grazing land | Spatially explicit dataset with a grid cell resolution of 10 x 10 km (ha km^-2^) | Velthuizen et al. (2007) |

**5. GHG emissions from feed scenarios for the whole dairy sector**

Table S6: GHG emissions per emission source and scenario as shown in Fig 5A. Emissions from each scenario are the aggregated emissions for all livestock production systems. Land to cultivate feeds include existing land allocated to cultivate Napier grass and maize, and converted grasslands, which result from land shortages.

|  | Emissions from enteric fermentation | Emissions from manure management | Emissions from feed cultivation | Converted grasslands | Emissions from land use change | Emissions from forest C change |
| --- | --- | --- | --- | --- | --- | --- |
|  | Mg CO_2_eq yr^-1^ | | | hectares | Mg CO_2_eq yr^-1^ | |
| Baseline | 28,764 | 3,890 | 3,234 | 0 | 0.00 | 782 |
| FeCo-Ya | 28,177 | 3,677 | 5,803 | 775,573 | 9,886 | 51 |
| FeCo-Yw50 | 28,212 | 2,880 | 10,260 | 308,346 | 3,941 | -693 |
| FeCo-Yw80 | 28,067 | 2,558 | 11,641 | 191,071 | 2,443 | -980 |
| FoFeCo-Ya | 22,701 | 7,824 | 8,862 | 1,685,556 | 21,397 | 407 |
| FoFeCo-Yw50 | 22,758 | 7,810 | 14,736 | 748,689 | 9,568 | -8 |
| FoFeCo-Yw80 | 22,760 | 7,811 | 16,867 | 518,162 | 6,623 | -449 |
| FoCo-Ya | 27,018 | 4,319 | 4,798 | 155,149 | 935 | -1,332 |

Table S7: GHG emission intensities (kg CO_2_eq kg fat and protein corrected milk (FPCM)^-1^) per emission source and scenario as shown in Fig 5B.

|  | Enteric fermentation | Manure management | Feed cultivation | Land use change | Forest C change | |
| --- | --- | --- | --- | --- | --- | --- |
| Baseline | 1.89 | 0.26 | 0.21 | 0.00 | 0.05 |  |
| FeCo - Ya | 1.28 | 0.17 | 0.26 | 0.45 | 0.00 |  |
| FeCo - Yw50 | 1.28 | 0.13 | 0.47 | 0.18 | -0.03 |  |
| FeCo - Yw80 | 1.28 | 0.12 | 0.53 | 0.11 | -0.04 |  |
| FoFeCo - Ya | 0.99 | 0.34 | 0.39 | 0.93 | 0.02 |  |
| FoFeCo - Yw50 | 0.99 | 0.34 | 0.64 | 0.42 | 0.00 |  |
| FoFeCo - Yw80 | 0.99 | 0.34 | 0.74 | 0.29 | -0.02 |  |
| FoCo - Ya | 1.22 | 0.20 | 0.22 | 0.04 | -0.06 |  |

**References**

Anindo, D. O., Said, A. N., & Lahlou-Kassi, A. (1994). Chemical Composition and Nutritive value of Feedstuffs for Ruminant Livestock in sub-Saharan Africa. Retrieved from International Livestock Centre for Africa website: http://192.156.137.110/ssafeed/

Bebe, B. O., Udo, H. M. J., & Thorpe, W. (2002). Development of smallholder dairy systems in the Kenya highlands. Outlook on Agriculture, 31(2), 113–120. https://doi.org/10.5367/000000002101293958

Brandt, P., Herold, M., & Rufino, M. C. (2018). The contribution of sectoral climate change mitigation options to national targets: a quantitative assessment of dairy production in Kenya. Environmental Research Letters, 13(3), 034016. https://doi.org/10.1088/1748-9326/aaac84

Castellanos-Navarrete, A., Tittonell, P., Rufino, M. C., & Giller, K. E. (2015). Feeding, crop residue and manure management for integrated soil fertility management - A case study from Kenya. Agricultural Systems, 134, 24–35. https://doi.org/10.1016/j.agsy.2014.03.001

Don, A., Schumacher, J., & Freibauer, A. (2011). Impact of tropical land-use change on soil organic carbon stocks - a meta-analysis. Global Change Biology, 17(4), 1658–1670. https://doi.org/10.1111/j.1365-2486.2010.02336.x

Government of Kenya. (2014). Livestock numbers and production dataset. Nairobi (Kenya): Ministry of Agriculture, Livestock and Fisheries.

Government of Kenya. (2015). Kenya’s Intended Nationally Determined Contribution (INDC). Retrieved from Ministry of Environment and Natural Resources website: http://www4.unfccc.int/ndcregistry/Pages/Party.aspx?party=KEN

Hengl, T., Heuvelink, G. B. M., Kempen, B., Leenaars, J. G. B., Walsh, M. G., et al. (2015). Mapping soil properties of Africa at 250 m resolution: Random forests significantly improve current predictions. PLoS ONE, 10(6). https://doi.org/10.1371/journal.pone.0125814

Herrero, M., Havlík, P., Valin, H., Notenbaert, A., Rufino, M. C., Thornton, P. K., et al. (2013). Biomass use, production, feed efficiencies, and greenhouse gas emissions from global livestock systems. Proceedings of the National Academy of Sciences of the United States of America, 110(52), 20888–20893. https://doi.org/10.1073/pnas.1308149110

Herrero, M., Thornton, P. K., Bernués, A., Baltenweck, I., Vervoort, J., van de Steeg, J. et al. (2014). Exploring future changes in smallholder farming systems by linking socio-economic scenarios with regional and household models. Global Environmental Change, 24(1), 165–182. https://doi.org/10.1016/j.gloenvcha.2013.12.008

Hickman, J. E., Huang, Y., Wu, S., Diru, W., Groffman, P. M., Tully, K. L., & Palm, C. A. (2017). Nonlinear response of nitric oxide fluxes to fertilizer inputs and the impacts of agricultural intensification on tropospheric ozone pollution in Kenya. Global Change Biology, 23(8), 3193–3204. https://doi.org/10.1111/gcb.13644

IPCC. (2006). IPCC Guidelines for National Greenhouse Gas Inventories, Prepared by the National Greenhouse Gas Inventories Programme (H. S. Eggleston, L. Buendia, K. Miwa, T. Ngara, & K. Tanabe, eds.). Retrieved from https://www.ipcc-nggip.iges.or.jp/public/2006gl/

IPCC. (2014). Summary for Policymakers. In: Climate Change 2013: The Physical Science Basis. Contribution of Working Group I to the Fifth Assessment Report of the Intergovernmental Panel on Climate Change [Eds Stocker, T.F., D. Qin, G.-K. Plattner, M. Tignor, S. K. Allen, J. Boschung, A. Nauels, Y. Xia, V. Bex and P.M. Midgley]. Retrieved from Intergovernmental Panel on Climate Change website: http://www.ipcc.ch/report/ar5/wg1

Katiku, P. N., Gachuiri, C. K., & Mbugua, P. N. (2011). Characterization of Dairy Cattle Farming in Mbeere District of Eastern Kenya. Livestock Research for Rural Development, 23(11). Retrieved from http://www.lrrd.org/lrrd23/11/kati23236.htm

Monfreda, C., Ramankutty, N., & Foley, J. A. (2008). Farming the planet: 2. Geographic distribution of crop areas, yields, physiological types, and net primary production in the year 2000. Global Biogeochemical Cycles, 22(1), 1–19. https://doi.org/10.1029/2007GB002947

Potter, P., Ramankutty, N., Bennett, E. M., & Donner, S. D. (2010). Characterizing the spatial patterns of global fertilizer application and manure production. Earth Interactions, 14(2). https://doi.org/10.1175/2009EI288.1

Robinson, T. P., Thornton, P. K., Franceschini, G., Kruska, R. L., Chiozza, F., et al. (2011). Global Livestock Production Systems. Retrieved from Food and Agriculture Organization of the United Nations, International Livestock Research Institute website: http://www.fao.org/docrep/014/i2414e/i2414e.pdf

Robinson, T. P., William Wint, G. R., Conchedda, G., Van Boeckel, T. P., Ercoli, V., et al. (2014). Mapping the global distribution of livestock. PLoS ONE, 9(5). https://doi.org/10.1371/journal.pone.0096084

Rosenstock, T. S., Mpanda, M., Pelster, D. E., Butterbach-Bahl, K., Rufino, M. C., et al. (2016). Greenhouse gas fluxes from agricultural soils of Kenya and Tanzania. Journal of Geophysical Research G: Biogeosciences, 121(6), 1568–1580. https://doi.org/10.1002/2016JG003341

Rufino, M. C., Herrero, M., Van Wijk, M. T., Hemerik, L., De Ridder, N., & Giller, K. E. (2009). Lifetime productivity of dairy cows in smallholder farming systems of the central highlands of Kenya. Animal, 3(7), 1044–1056. https://doi.org/10.1017/S1751731109004248

Rufino, M. C., Tittonell, P., van, W., Castellanos-Navarrete, A., Delve, R. J., et al. (2007). Manure as a key resource within smallholder farming systems: Analysing farm-scale nutrient cycling efficiencies with the NUANCES framework. Livestock Science, 112(3), 273–287. https://doi.org/10.1016/j.livsci.2007.09.011

van Ittersum, M. K., Cassman, K. G., Grassini, P., Wolf, J., Tittonell, P., & Hochman, Z. (2013). Yield gap analysis with local to global relevance—A review. Field Crops Research, 143, 4–17. https://doi.org/10.1016/j.fcr.2012.09.009

Velthuizen, H. van, Huddleston, B., Fischer, G., Salvatore, M., et al. (2007). Mapping biophysical factors that influence agricultural production and rural vulnerability. Retrieved from Food and Agriculture Organization of the United Nations website: http://www.fao.org/docrep/pdf/010/a1075e/a1075e00.pdf

Weiler, V., Udo, H. M. J., Viets, T., Crane, T. A., & De, Boer. (2014). Handling multi-functionality of livestock in a life cycle assessment: The case of smallholder dairying in Kenya. Current Opinion in Environmental Sustainability, 8, 29–38. https://doi.org/10.1016/j.cosust.2014.07.009
